# A Highly Dense Genetic Map for *Ginkgo biloba* Constructed Using Sequence-Based Markers

**DOI:** 10.3389/fpls.2017.01041

**Published:** 2017-06-15

**Authors:** Hailin Liu, Fuliang Cao, Tongming Yin, Yingnan Chen

**Affiliations:** Co-Innovation Center for Sustainable Forestry in Southern China, College of Forestry, Nanjing Forestry UniversityNanjing, China

**Keywords:** *Ginkgo biloba*, specific-locus amplified fragment sequencing, megagametophyte, high density, linkage map

## Abstract

*Ginkgo biloba* L. is a well-known living gymnosperm fossil that has medicinal and ornamental value. In this study, a high density genetic map was constructed with megagametophytes of 94 seeds from a single *Ginkgo* tree by employing the specific-locus amplified fragment (SLAF) sequencing technique. The average sequencing depth was 11.20×, which yielded 538,031 high-quality SLAFs. Among these SLAFs, 204,361 were heterozygous in the maternal tree and segregated in the progeny. The established map contained 12,263 SLAFs that were assigned to 12 linkage groups (LGs). The number of LGs on this map equaled the number of chromosomes in *Ginkgo*. The total map length was 1,671.77 cM, with an average distance of 0.89 cM between adjacent marker bins. Map evaluation based on the haplotype map and the heat map validated the high quality of the established map. Because *Ginkgo* is an economically and biologically important tree, strenuous efforts have been exerted to sequence its genome. This new map, built using sequence-based markers, will serve in the future as a fundamental platform for anchoring sequence assemblies along *Ginkgo* chromosomes. This map also provides a desirable platform for various genetic studies of *Ginkgo*, including gene/quantitative trait locus mapping and marker-aided selection.

## Introduction

*Ginkgo biloba* L., or maidenhair tree, is one of the oldest “living fossils.” The first appearance of its crown was found approximately 280 million years ago in the early Permian period according to the fossil records (Zhou and Zheng, 2003; Gong et al., 2008). *Ginkgo* tree morphology has remained virtually unchanged for millions of years (Galián et al., 2012). In addition, *G. biloba* is the sole living member of the Ginkgoalean clade. This species is a precious link between primitive non-flowering plants and advanced seed plants (Hori et al., 2012). The *Ginkgo* tree, which originated in China (Gong et al., 2008), has been used in traditional Chinese medicine for thousands of years (Goh and Barlow, 2002). *G. biloba* has now been introduced to many areas of the world because of its high ornamental value, immense environmental adaptability and strong resistance to almost all plant pests and diseases (Major, 1967). *Ginkgo* leaf extracts are also widely used in herbal remedies in Europe and as a dietary supplement in the United States (Ahlemeyer and Krieglstein, 2003).

Serious efforts are currently being made to unravel the mystery of the *Ginkgo* genome (Guan et al., 2016), and one of the best platforms to achieve this goal is genetic map, as genetic mapping is a robust tool used in map-based cloning, QTL mapping and sequencing scaffold allocation (Collard et al., 2005; Heesch et al., 2010). Several types of markers have been previously developed for *Ginkgo* genetic studies. These include isozyme (Wu et al., 1992; Tsumura and Ohba, 1997), RAPD (Grattapaglia and Sederoff, 1994; Lynch and Milligan, 1994; Kuddus et al., 2002; Fan et al., 2004), ISSR (Prevost and Wilkinson, 1999; Joshi et al., 2000; Ge et al., 2002, 2003) and AFLP (Wang et al., 2008) markers. Lin et al. (2011) recently developed 204 EST – SSR markers in *G. biloba* using Roche 454 sequencing, while Han et al. (2015) generated more than 4,000 SSRs based on a *Ginkgo* cDNA library. With the availability of these marker resources, several genetic maps have been reported for *Ginkgo*. Tan (1997) constructed the first genetic linkage map of *G. biloba* containing 62 RAPD markers, with a total length of 829.10 cM. Gui (2004) build a genetic map of *G. biloba* covering 1742.20 cM based on RAPD and ISSR markers. However, the above maps were all constructed with anonymous markers. Until very recently, Tao (2014) developed a linkage map containing a limited number of non-anonymous markers. To facilitate genome sequencing of *Ginkgo*, a highly dense linkage map constructed with sequence-based markers is strongly needed.

In recent years, advances in next-generation sequencing technologies have enabled the simultaneous generation of an enormous number of sequence-based markers. These markers are highly desirable for genetic map construction because of their abundance, uniform distribution, and cost-effectiveness (Ganal et al., 2009). RAD-seq is one of several high-throughput techniques that have facilitated large-scale marker production. SLAF-seq, which is based on RAD-seq, was developed as a high-resolution strategy for *de novo* SNP discovery with good repeatability (Sun et al., 2013; Qi et al., 2014). This approach has been successfully applied for genetic map construction in many plant species, such as soybean, orchard grass and willow (Qi et al., 2014; Zhang et al., 2016; Zhao et al., 2016).

In this study, a high-density genetic map for *G. biloba* was constructed using SLAF-seq. To our knowledge, this is the densest genetic map generated for *G. biloba* with sequence-based markers. This platform is not only useful for locating sequence assemblies along the chromosomes of *Ginkgo*, but is also highly applicable to the identification of QTLs and genes underlying agronomic traits of this living fossil plant, including outstanding tolerance to abiotic stress, high resistance to plant diseases and pests, and biological synthesis of multiple medicinal ingredients.

## Materials and Methods

### Plant Materials and DNA Extraction

Mature seeds were collected from a female *Ginkgo* tree on the campus of Nanjing Forestry University in October 2014. The megagametophyte tissues of 94 seeds were carefully isolated by removing the endotesta and embryo. Total DNA was extracted from each megagametophyte using a DNeasy Plant Mini kit (Qiagen, Hilden, Germany). DNA concentration was quantified by using NanoDrop 2000 (Thermo Scientific, Waltham, MA, United States) and stored at -80°C until use.

### SLAF Library Preparation and Sequencing

Specific-locus amplified fragment library preparation and sequencing were performed according to Sun et al. (2013) with minor modifications. First, 320 ng genomic DNA from each megagametophyte was digested with 5 U *EcoRI* and 5 U *MseI* (New England Biolabs, NEB) at 37°C for 6 h, then restriction-ligation reactions were heat-inactivated at 65°C for 20 min with T4 DNA ligase (NEB), ATP (NEB), *EcoRI*-adapter and *MseI*-adapter. The mixture was finally incubated at 20°C for 12 h. Preamplification reactions (40 μL) were performed with 5 μL of the diluted restriction-ligation mixture using a pair of primers with one selective nucleotide each (*EcoRI*+A; *MseI*+C). Thermocycling conditions, optimized to promote specificity, were as follows: 20 cycles of 94°C for 30 s, 56°C for 60 s, and 72°C for 60 s. The PCR products were purified by using E.Z.N.A. Cycle Pure kit (Omega), then pooled and incubated at 37°C with *MseI, EcoRI*, T4 DNA ligase, ATP and Solexa adapter (Illumina). After incubation, the pooled samples were purified using a Quick Spin column (Qiagen), and electrophoresed on a 2% agarose gel.

DNA fragments between 500 and 550 bp (SLAFs) were excised and isolated using a Gel Extraction kit (Qiagen). The isolated fragments were then subjected to PCR amplification with Phusion Master Mix (NEB) and Solexa amplification primer mix (Illumina) for barcoding according to the Illumina sample preparation guide. After gel purification, SLAFs between 114 and 464 bp were isolated and diluted for paired-end sequencing (2 × 125 bp) on an Illumina-HiSeq 2500 platform (Illumina) at Beijing Biomarker Technologies Corporation^1^. Raw reads were assigned to different samples according to the barcode sequences.

### Sequence Clustering and Association Analysis

Single nucleotide polymorphism identification and genotyping were performed as described by Sun et al. (2013) with a few modifications. After filtering of low-quality reads (quality score <30, indicating a 0.10% chance of an error and thus 99.90% confidence), all paired-end reads with clear index information were grouped into clusters based on sequence similarity using BLAT (Kent, 2002). Nearly identical reads assigned in a single group were considered as one SLAF locus (Sun et al., 2013). Alleles at each locus were subsequently defined by MAF evaluation. SLAF tags with markedly lower MAF values were corrected to the most similar genotype to improve data accuracy.

Because the megagametophyte tissue of *G. biloba* is haploid, heterozygous SLAFs in the maternal tree were expected to contain two allelic tags, which could be recorded as an ‘hk × hk’ segregation matrix. For using in genetic map construction, we selected the identified ‘hk × hk’ SLAFs that had an average depth >2, contained fewer than five SNPs, and were present in at least 70% of progeny.

### Linkage Analysis and Genetic Map Construction

Specific-locus amplified fragments were assigned to different LGs based on the pairwise modified logarithm of odds (MLOD) score. Markers with MLOD score < 5 were abandoned prior to ordering. HighMap software was used to order the SLAF markers and to correct genotyping errors within LGs (Liu et al., 2014). Briefly, recombination frequencies and MLOD scores were calculated by two-point analysis. Then, a combination of enhanced Gibbs sampling, spatial sampling and simulated annealing algorithms was used to perform an iterative process of marker ordering. SMOOTH algorithm was adopted to correct the genotyping errors, and the *k*-nearest neighbor algorithm was applied to impute missing genotypes. Markers showing significant segregation distortion (*P* < 0.01) were excluded from map construction. Map distances were estimated using the Kosambi mapping function. Haplotype map and heat map were used to evaluate the quality of the genetic map (West et al., 2006).

## Results

### Analysis of SLAF-seq Data and SLAF Markers

**FIGURE 1:**
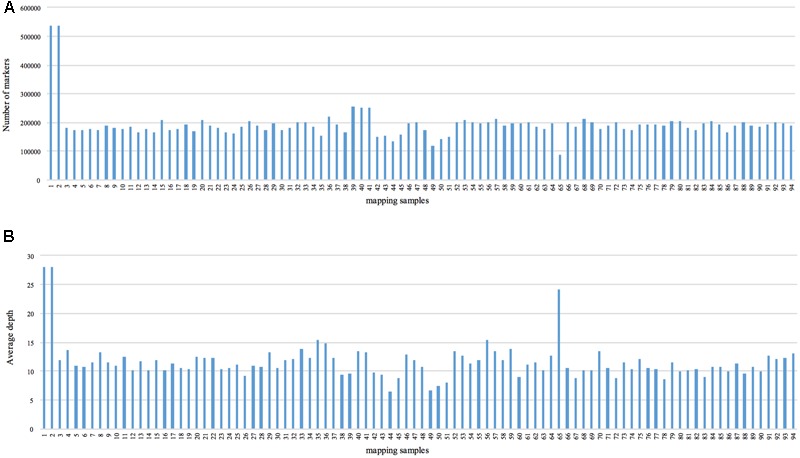
Number of markers and average sequencing depth of each progeny. The *x*-axes in **(A,B)** indicate the identity of the mapping samples, and the *y*-axes indicate the number of markers **(A)** and average depth **(B)**.

We obtained approximately 120 Gb of data containing 929.43 M raw reads. The length of each read was 126 bp, with a Q30 ratio of 89.21% and a guanine-cytosine content of 42.60% (**Table 1**). After removing low-quality reads, 538,031 SLAFs were recovered, with the number of SLAFs varying from 87,476 to 538,031 among the different progeny (**Figure 1**). The average sequencing depth of these SLAFs was 11.20×, with coverage ranging from 6.52× to 28.01× (**Figure 1**). Among the identified SLAFs, 204,361 (37.98%) were heterozygous in the maternal tree (**Table 2**), and 201,667 (37.48%) segregated in a 1:1 ratio in the progeny. The raw data of SLAF-seq have been submitted to NCBI SRA database under the accession number SRP105402.

**Table 1:** Summary of SLAF-seq results obtained using megagametophytes of 94 seeds from a single *Ginkgo biloba* tree.

Total number of reads (*M*)	929, 43
Q30 percentage (%)	89.21
GC percentage (%)	42.60
Total SLAFs	538, 031
Mean sequencing depth of offsprings	11.20×
Mapped markers	12, 263
Mean sequencing depth of the SLAFs on the map	32.37×

**Table 2:** Classification of SLAF markers.

Type	Polymorphic SLAFs	Non-polymorphic SLAFs	Total SLAFs
Number	204361	333670	538031
Depth	190171039	35367625	225538664
Percentage (%)	38	62	100

### High-Density Genetic Linkage Map Construction

**FIGURE 2:**
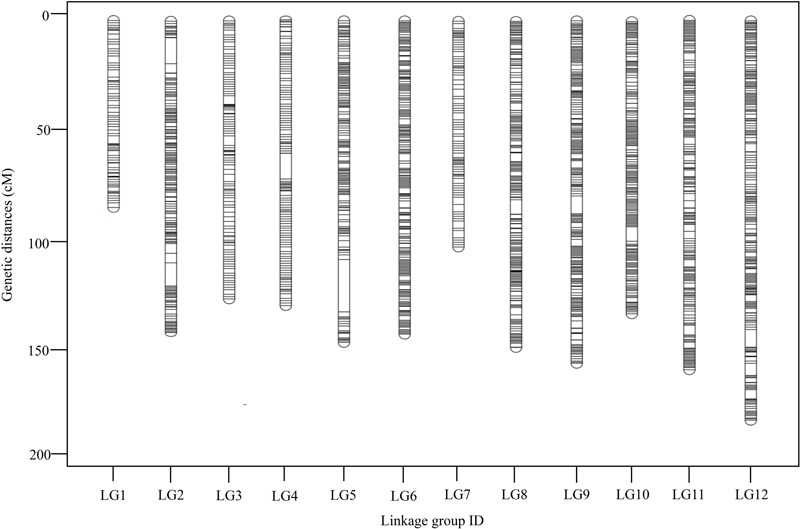
Distribution of SLAFs along each of the linkage group of *G. biloba*. A black line indicates a SLAF marker.

**FIGURE 3:**
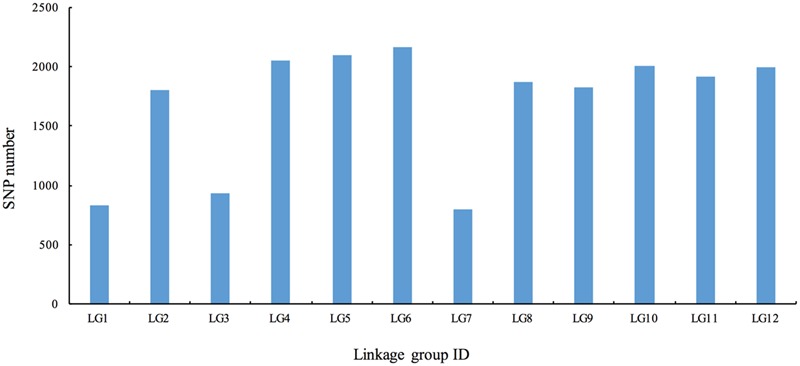
The number of single nucleotide polymorphism (SNP) markers on the each of the linkage group.

To ensure the quality of the genetic map, SLAFs meeting any of the following criteria were excluded: (1) number of SNPs >5; (2) integrity <70%; (3) significantly distorted segregation (*P* < 0.01). A total of 13,180 SLAF markers were retained, of which 93.04% (12,263) were assigned to 12 LGs (**Figure 2** and **Supplementary Figure S1**). The mean sequencing depth of the mapped SLAFs was 32.37× (**Table 1**), and the number of SLAFs in each LG ranged from 483 to 1,339 (**Table 3**). Overall, the mapped SLAFs included 20,297 SNP markers. The number of mapped SNPs per LG ranged from 802 to 2,169 (**Figure 3**). The established map consisted of 1,961 marker bins; it covered a genetic distance of 1671.77 cM, with LG sizes ranging from 86.1 to 183.80 cM. The average distance between marker bins was 0.89 cM (**Table 3**). LG6 and LG10 had the highest marker density, 0.70 cM between marker bins, whereas LG7 had the lowest density of 1.22 cM between marker bins on average.

**Table 3:** Characteristics of the 12 linkage groups of *G. biloba*.

Linkage group	Loci number			Markers number	Total distance /cM	Average distance /cM	Gaps^∗∗^<5 cM/%	Max Gap/cM
	Bin^∗^ (Marker)	Singleton	Total (framework)					
LG1	41 (444)	51	92	495	86.11	0.92	100	3.44
LG2	79 (999)	84	163	1,083	143.30	0.87	98.77	11.91
LG3	73 (535)	44	117	579	128.00	1.08	100	4.45
LG4	88 (1243)	30	118	1,273	131.45	1.10	99.15	11.79
LG5	83 (1159)	73	156	1,232	148.01	0.94	99.35	24.14
LG6	99 (1232)	107	206	1,339	144.19	0.70	100	3.30
LG7	67 (467)	16	83	483	103.92	1.22	100	3.30
LG8	94 (980)	107	201	1,087	150.03	0.74	99.50	6.72
LG9	102 (983)	111	213	1,094	157.59	0.74	99.53	8.04
LG10	97 (1112)	94	191	1,206	134.53	0.70	99.47	6.65
LG11	98 (1084)	99	197	1,183	160.84	0.81	100	4.45
LG12	105 (1090)	119	224	1,209	183.80	0.82	99.10	8.06
Total	1026	935 (47.70%)	1961	12,263	1,671.77	0.89	99.57	–

*^∗^Marker pairs with zero recombination on each linkage group, considered to belong to the same genetic bin; ^∗∗^the percentage of locus intervals where the distance between adjacent loci was smaller than 5 cM.*

**FIGURE 4:**
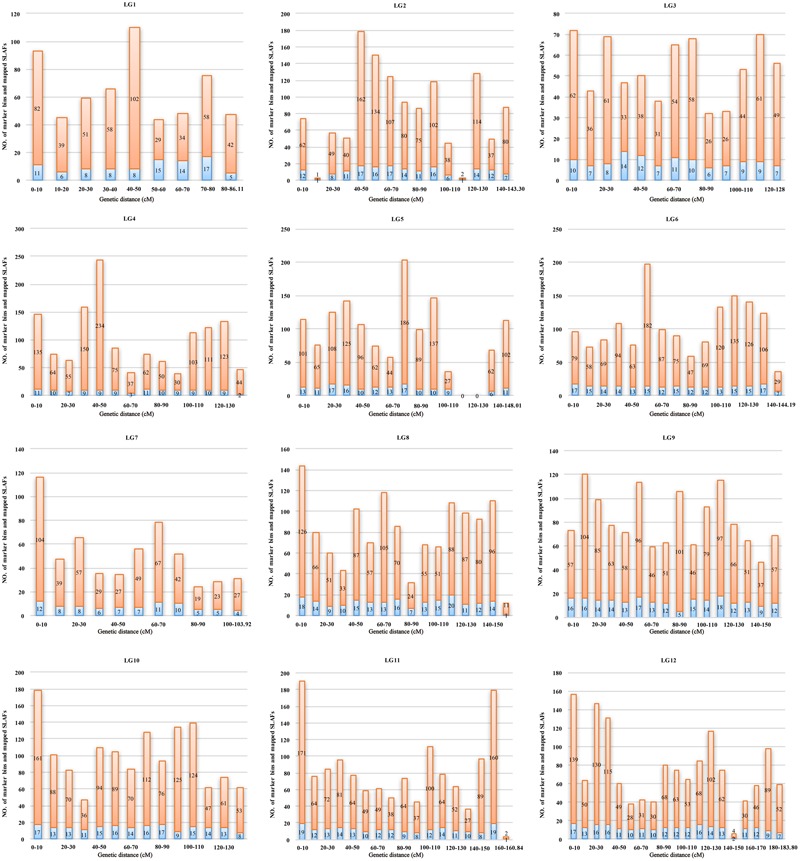
Distribution of marker bins and SLAFs in the 10 cM sliding windows among each linkage group. The number of marker bins and the number of mapped SLAFs were indicated with blue and orange bars, respectively.

Marker distribution was analyzed by counting the number of loci and all mapped markers (including bins and singletons) by sliding 10-cM windows along each LG (**Supplementary Table S1**). According to this analysis, the mapped markers were distributed relatively uniformly along each LG. The ‘Gap < 5 cM’ value, which indicated the degree of linkage between adjacent loci among LGs, ranged from 98.77 to 100%, with an average of 99.57%. Only nine gaps >5 cM were detected on seven LGs. Notably, a large gap was present on LG5 spanning a genetic length of 24.14 cM (**Figure 4**). Possible explanations for this gap are that the corresponding region is a restriction desert for *EcoRI*/*MseI* or alternatively it may be associated with a recombination hotspot. The region with the highest marker density, on LG4, contained 243 SLAFs within a 10-cM interval. According to the 10-cM sliding window analysis, the average number of marker bins ranged from 8 to 14, with the average number of SLAFs varying from 44 to 91 (**Supplementary Table S1**).

### Segregation Distortion of Mapped SLAFs

Among the 12,263 mapped SLAFs, 168 showed segregation distortion at a significance level of *P* < 0.05 (**Supplementary Table S2**). Segregation-distorted markers were observed in clusters on LG1, LG5 and LG7, with 1 to 117 segregation-distorted SLAFs in each cluster (**Table 4** and **Supplementary Table S3**). The largest segregation distortion cluster was observed on LG7 and contained 24.22% of the markers mapped to this LG (**Table 4**).

**Table 4:** Distribution of segregation distortion markers.

Linkage group	All markers		Segregation distortion^∗^ markers		Frequency of segregation distortion marker /%	
	Number	Percentage/%	Number	Percentage/%	
LG1	495	4.04	50	29.76	10.10
LG2	1,083	8.83	0	0.00	0.00
LG3	579	4.72	0	0.00	0.00
LG4	1,273	10.38	0	0.00	0.00
LG5	1,232	10.05	1	0.60	0.80
LG6	1,339	10.92	0	0.00	0.00
LG7	483	3.94	117	69.64	24.22
LG8	1,087	8.86	0	0.00	0.00
LG9	1,094	8.92	0	0.00	0.00
LG10	1,206	9.83	0	0.00	0.00
LG11	1,183	9.65	0	0.00	0.00
LG12	1,209	9.86	0	0.00	0.00
Total	12263	–	168	–	–

*^∗^The significance level of segregation distortion is 0.01 < *P* < 0.05.*

### Evaluation of the Genetic Map

A haplotype map can reveal double crossovers within a short genetic distance, which are closely associated with genotyping errors (Qi et al., 2014). In this study, haplotype maps were generated for each of the 94 progeny using the 12,263 SLAF markers (**Supplementary Figure S2**). Recombination events and missing data for each individual could be clearly visualized with the haplotype maps. As inferred from these maps, the ratio of double crossovers was less than 0.20%, while the percentage of missing data on each LG was less than 0.27% (**Table 5**). The established map is thus of high quality and consequently reliable for future application to studies of different genetic aspects of *Ginkgo*.

**Table 5:** Percentages of double crossovers and missing data in each linkage group.

Linkage group ID	Singleton percent (%)	Missing percent (%)
LG1	0.20	0.27
LG2	0.00	0.13
LG3	0.00	0.40
LG4	0.00	0.02
LG5	0.08	0.09
LG6	0.00	0.15
LG7	0.00	0.10
LG8	0.00	0.13
LG9	0.00	0.14
LG10	0.08	0.14
LG11	0.08	0.13
LG12	0.00	0.13

Heat maps derived from pairwise recombination values of mapped SLAFs can also be used to evaluate the quality of an established map (**Supplementary Figure S3**), especially with respect to potential ordering errors. In the generated heat maps, an obvious trend was observed, with pairwise linkage generally weakening as genetic distance increased between mapped markers. This pattern indicated that the markers in each LG were well ordered.

## Discussion

Linkage map is one of the basic platforms for research on the genetics and genomics of focal organisms (Liu et al., 2014). Although mapping populations for higher plants should ideally be derived from crosses between inbred lines, such populations are impossible or very difficult to obtain for forest trees because of their high genetic load and time constraints. Seeds of gymnosperms carry maternally inherited haploid megagametophytes, which are as efficient as inbred lines in terms of map construction (Wu et al., 1999). Haploid megagametophytes have therefore been used to establish linkage maps for many coniferous species, such as *Picea glauca* (Tulsieram et al., 1992), *Picea abies* (Binelli and Bucci, 1994), *Pinus pinaster* (Plomion et al., 1995) and *Pinus taeda* (Wilcox et al., 1996). Gui (2004) built a linkage map for *Ginkgo* using megagametophytes from a single tree as a mapping population, but the map only contained a few anonymous markers.

Here, we have established a high-density genetic map for *Ginkgo* that comprises 12 LGs. The number of LGs is equivalent to the haploid chromosome number of *Ginkgo* (Ishikawa, 1910; Murray, 2013). Gui (2004) also build a *Ginkgo* genetic map with 12 LGs. However, the current genetic map contains a much larger number of markers (∼74.77-fold) than that developed by Gui (2004). It is noteworthy that the two maps span a similar genetic length, indicating the *Ginkgo* map established in this study is highly saturated. The density of this map is higher than that of any other maps reported for gymnosperms thus far. For instance, marker densities in the most recently reported gymnosperm genetic maps are 2.80 cM in *P. abies* (Lind et al., 2014) and 1.77 cM in *Callitris glaucophylla* (Sakaguchi et al., 2015). The haploid genome size of *G. biloba* has been estimated to be 10.61 Gb (Guan et al., 2016). Thus the average physical/genetic distance ratio is approximately 6.35 Mb/cM for the *Ginkgo* genome. This value is smaller than that of sequenced conifers such as *P. abies* (10.40 Mb/cM; Lind et al., 2014) and *P. taeda* (17.92 Mb/cM; Eckert et al., 2009; Zimin et al., 2014). The genome of *Ginkgo* has been sequenced (Guan et al., 2016) and continuing efforts are exerted on improving the genome assembly of *Ginkgo* with the PacBio sequences. Our map provides a desirable platform for reconstructing the *Ginkgo* chromosomes with the improved sequence assemblies in future.

Segregation distortion is a phenomenon frequently observed in mapping studies of different organisms (Ma et al., 2015). Segregation distortion might associate with some non-biological factors, such as finite sampling, sampling errors and genotyping errors (Kuang et al., 1999; Alheit et al., 2011), especially if the segregation distortion markers are randomly dispersed across the map and do not aggregate into clusters (Liu et al., 2011). It is noteworthy that the mapped segregation distorted markers are clustered on LG1and LG7 in this study. The observation of large clusters of segregation distorted markers suggested that their occurrence could not be explained by non-biological factors alone (Li et al., 2012), and biological factors were more likely to be the ultimate causes giving rise to segregation distortion observed in this study.

To our knowledge, this map is the first one conducted for *Ginkgo* using high-throughout sequencing technology. We used the recently developed SLAF-seq method to conduct *de novo* SNP discovery and large-scale genotyping. Despite the rapid development of sequencing technologies, population-scale whole-genome sequencing is still cost prohibitive (Sun et al., 2013). SLAF-seq combined with reduced genome amplicons provides a cost-effective alternative for population-level SNP recovery, especially for plants with gigantic genomes like *Ginkgo*. Furthermore, compared with RAD-seq data, the paired-end reads obtained by SLAF-seq can increase marker specificity, accuracy and efficiency with double barcode system (Shan et al., 2015). SLAF-seq methods have been applied in a variety of plant species, including sesame (Zhang et al., 2013), kiwifruit (Huang et al., 2013), soybean (Han et al., 2015), rice (Xu et al., 2015), tea plants (Ma et al., 2015) and Chinese fir (Su et al., 2016). In this study, the SLAF technique was successfully applied for *Ginkgo* genetic map construction, thereby providing a useful platform for various future genetic studies of this living fossil plant.

## Conclusion

A total of 538,031 high-quality SLAF markers were developed. Among these, 204,361 polymorphic markers were identified, with a polymorphism rate of 37.98%. Among the generated SLAF markers, 12,263 met the requirements for constructing a genetic map. These SLAF markers were distributed uniformly on each LG. Finally, 20,297 SNP markers were ultimately integrated into the genetic linkage map. SLAF-seq can thus be successfully used for large-scale SNP identification and genotyping in *G. biloba*.

The genetic map developed in this study is the first SNP-based reference map of *G. biloba* and is also the densest map to date. This high-density map can serve as a valuable tool for QTL fine mapping and positional cloning of candidate genes for economically important traits of *Ginkgo*. The map should also aid molecular breeding and anchoring of scaffolds to facilitate whole-genome sequencing projects for this species.

## Author Contributions

HL conducted the experiments and prepared the manuscript. FC helped in the design of the study and the collection of plant materials. YC and TY participated in the design and helped to draft the manuscript. All authors read and approved the final manuscript.

## Conflict of Interest Statement

The authors declare that the research was conducted in the absence of any commercial or financial relationships that could be construed as a potential conflict of interest.

## Footnotes

1 http://www.biomarker.com.cn

## Supplementary Material

FIGURE S1 Genetic map of *G. biloba*.

FIGURE S2 Haplotype map for each linkage group by each individual. Different individuals in rows were spaced by blank line, markers on the right were listed coordinate to the order in the linkage map. The swift of green and blue colors indicates the recombination events. The gray color represents missing marker.

FIGURE S3 Heat map of each linkage group by pair-wise markers. Each cell represents the recombination rate of pair-wise markers. Yellow color indicates strong linkage, and changes of colors from yellow to purple indicating weaker linkage.

TABLE S1 The number of loci and all the mapped markers (including bins and singletons) by sliding 10 cM windows along each LG.

TABLE S2 The list of segregation distorted SLAFs.

TABLE S3 The distribution of the segregation distorted SLAFs in each LG.

## Abbreviations

AFLP: amplified fragment length polymorphism
EST: expressed sequence tag
ISSR: inter-simple sequence repeat
LG: linkage group
MAF: minor allele frequency
QTL: quantitative trait locus
RAD-seq: restriction site-associated DNA sequencing
RAPD: random amplified polymorphic DNA
SLAF-seq: specific-locus amplified fragment sequencing
SNP: single nucleotide polymorphism
SSR: simple sequence repeat
