# Supplementary figures and images for: A Highly Dense Genetic Map for Ginkgo biloba Constructed Using Sequence-Based Markers
Source: Front Plant Sci. 2017 Jun 15;8:1041. doi: 10.3389/fpls.2017.01041 (PMC5471298; doi:10.3389/fpls.2017.01041)

LG 1

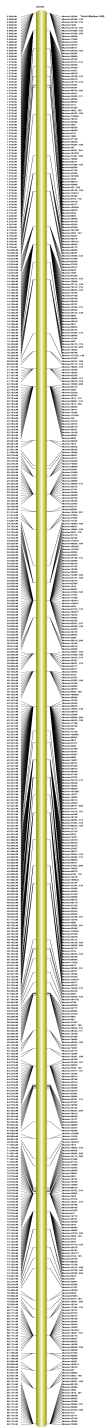

LG 2

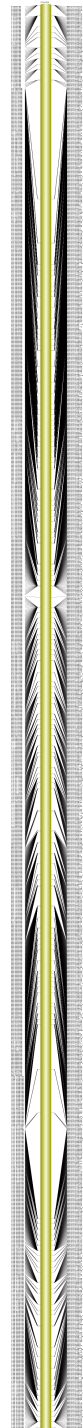

LG 3

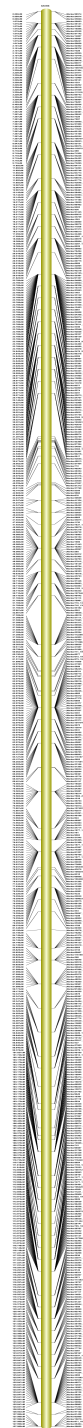

LG 4

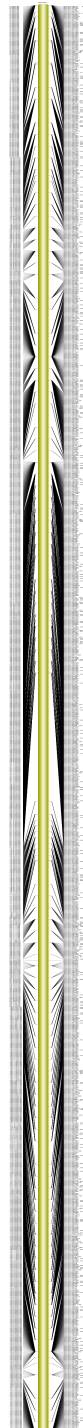

LG 5

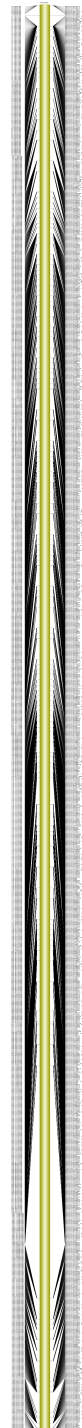

LG 6

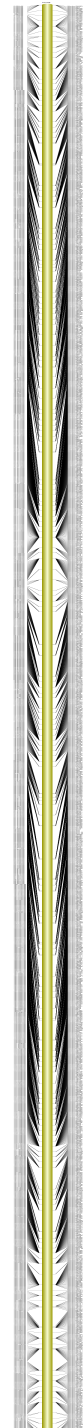

LG 7

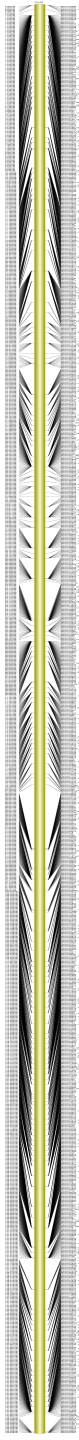

LG 8

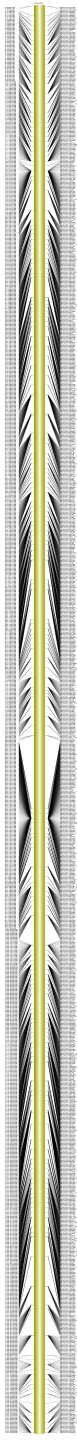

LG 9

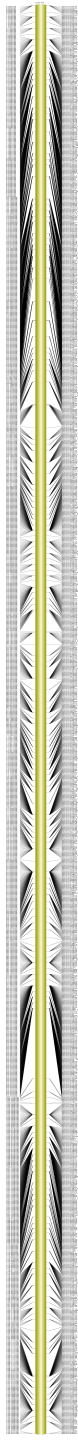

LG 10

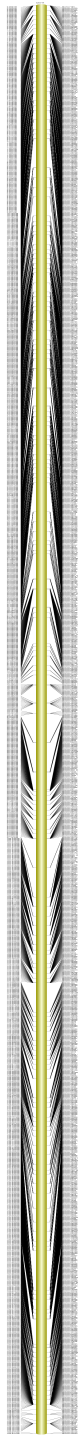

LG 11

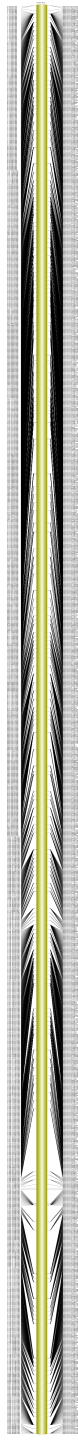

LG 12

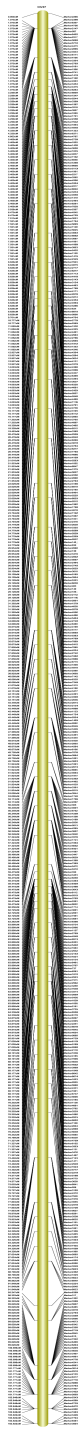

Supplement: FIGURE S1 — Genetic map of G. biloba. [file Image_1.PDF]

LG 1

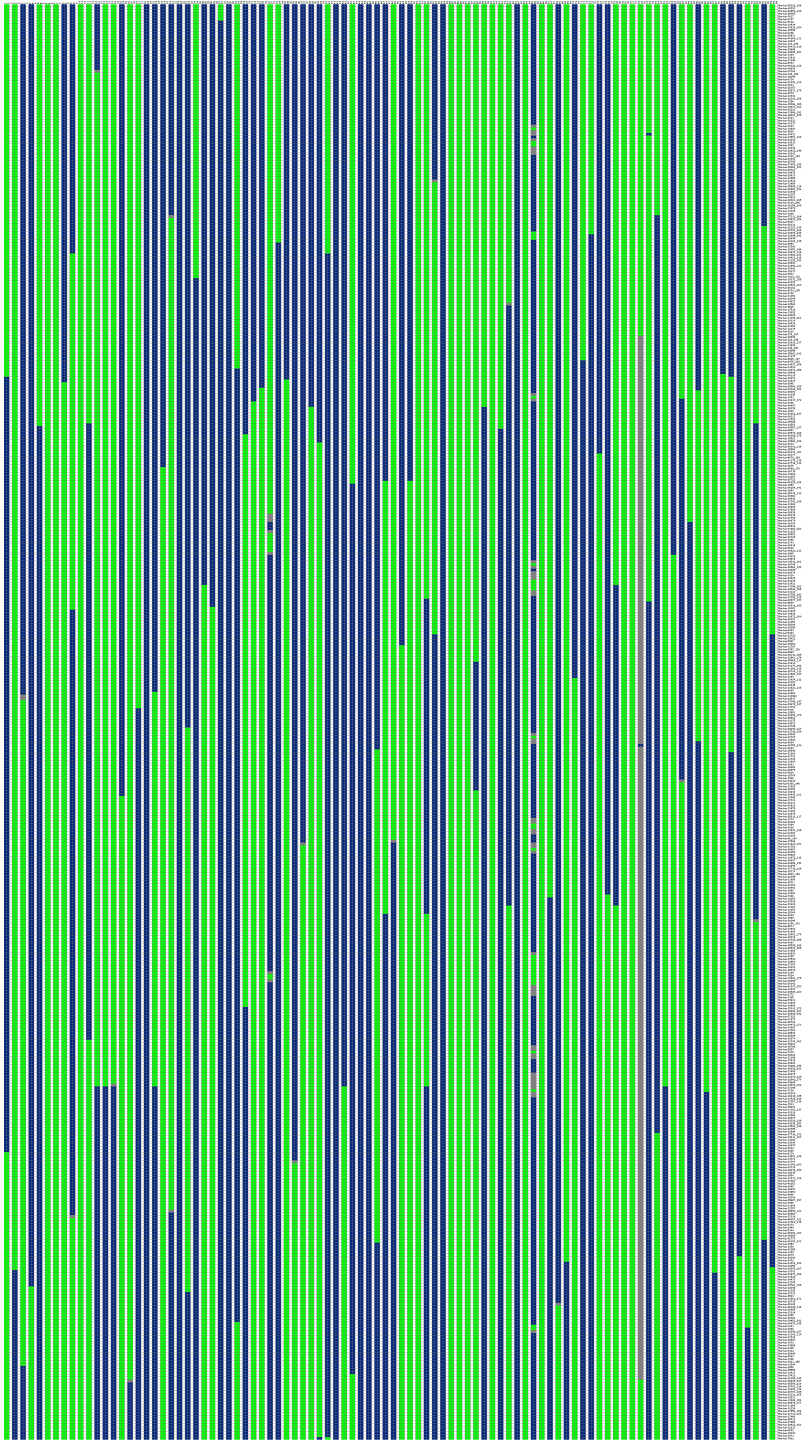

LG 2

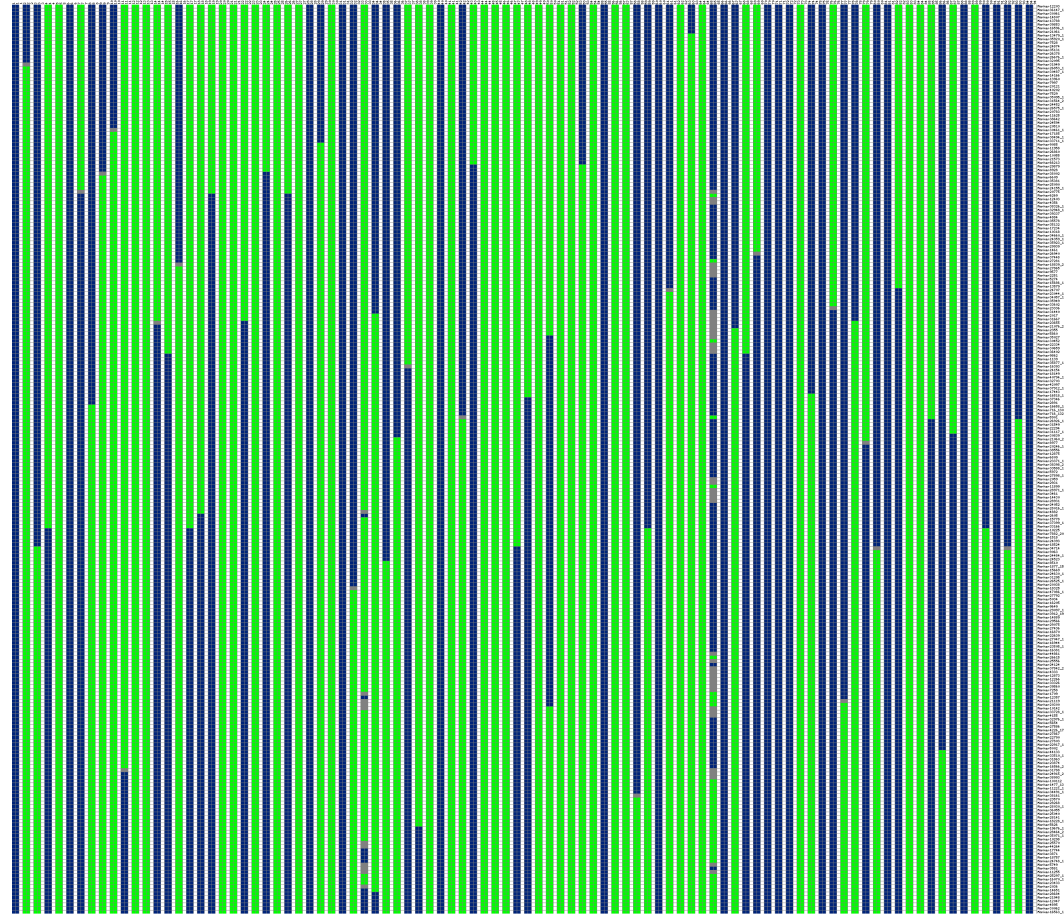

LG 3

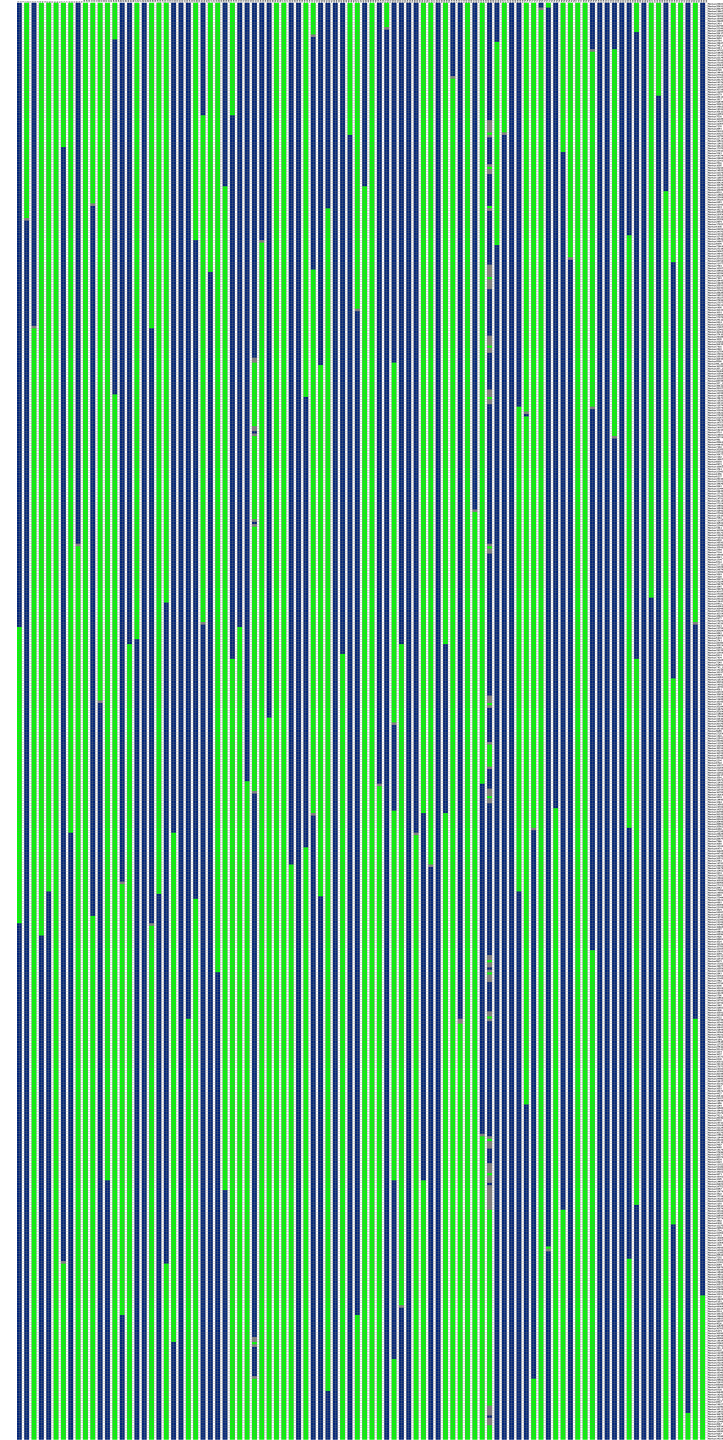

## LG 4

LG 5

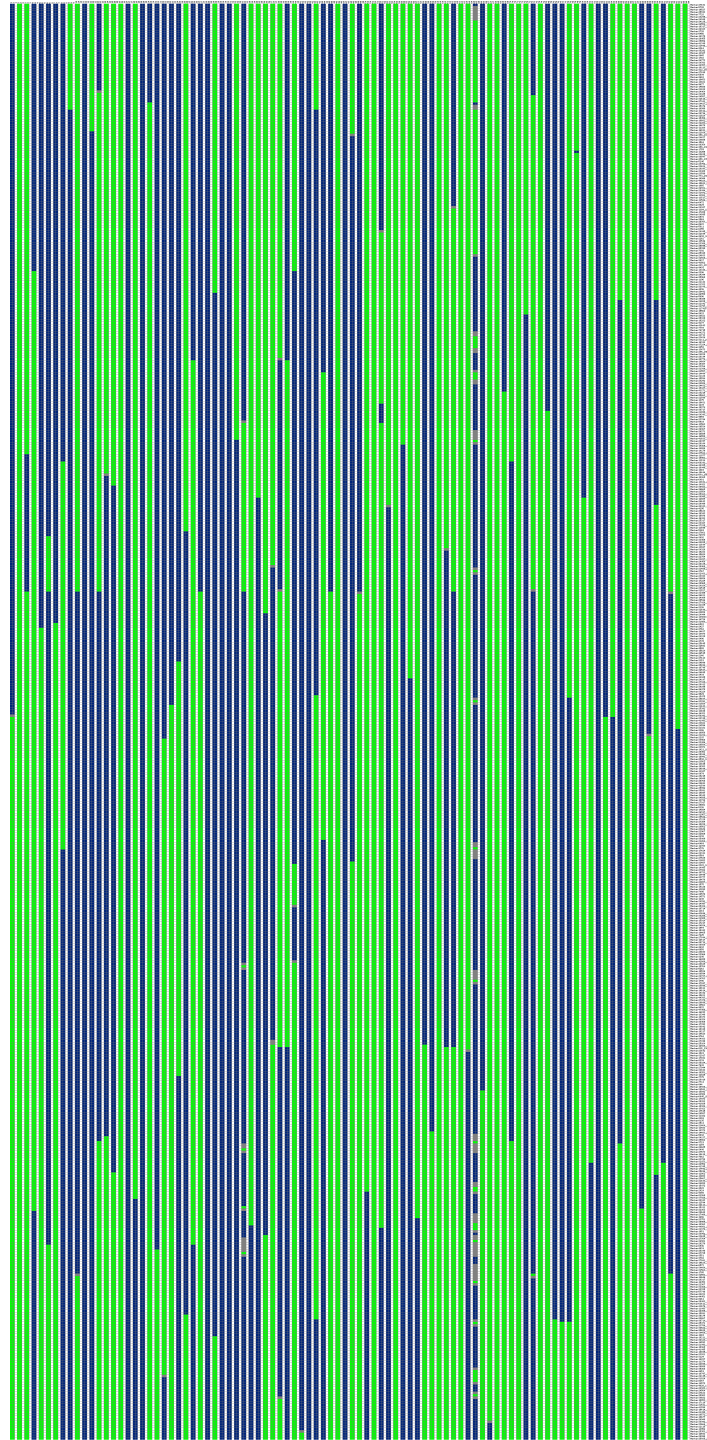

LG 6

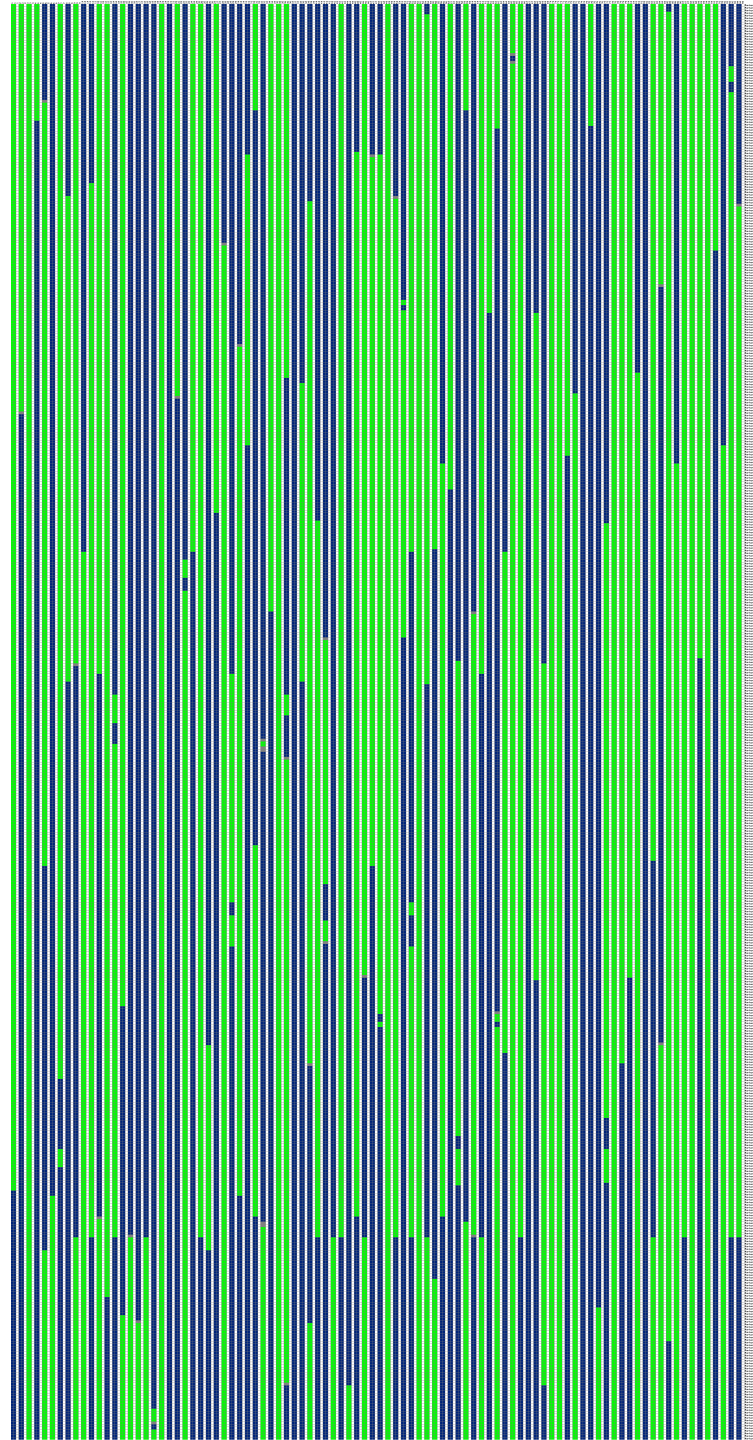

LG 7

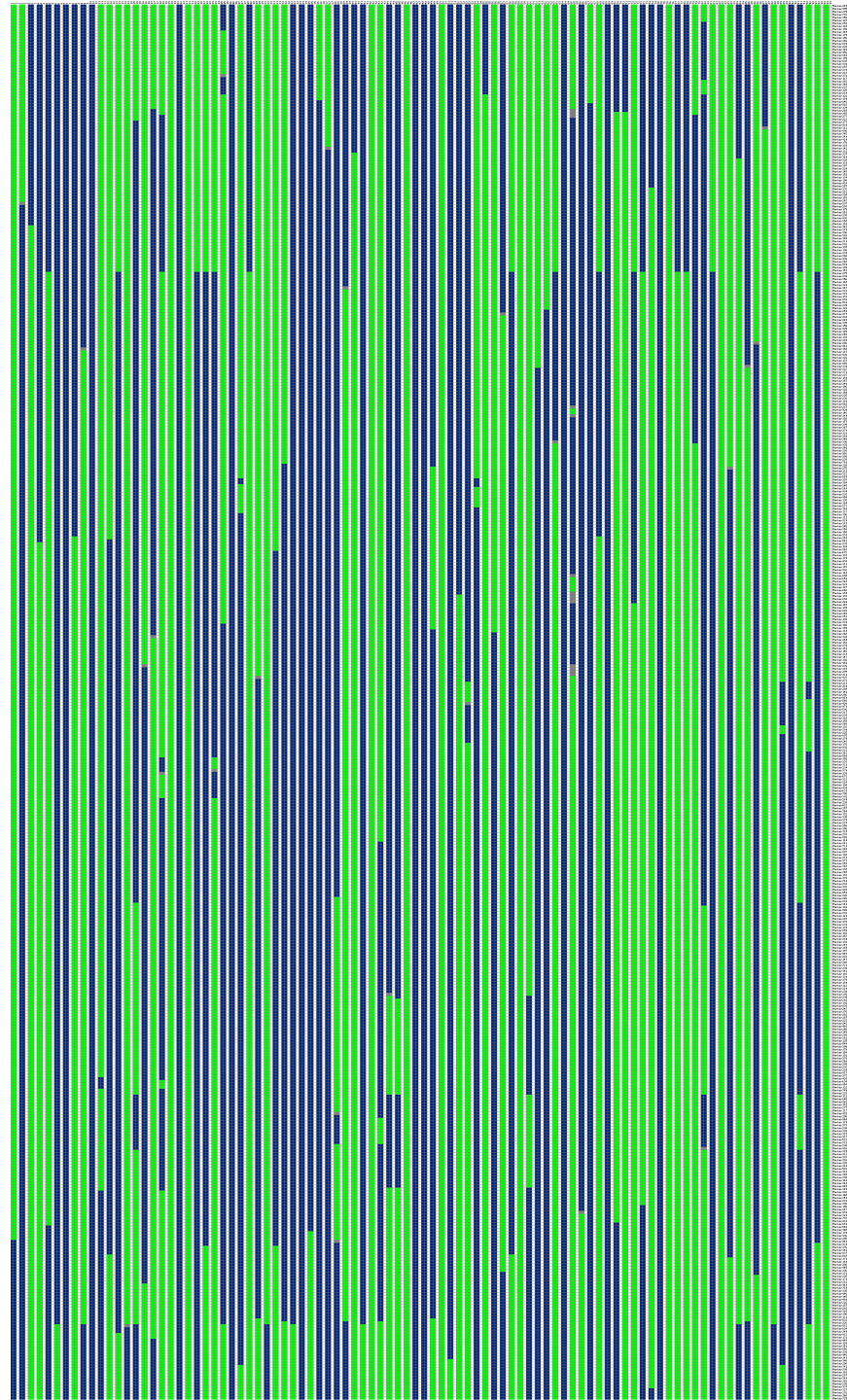

## LG 8

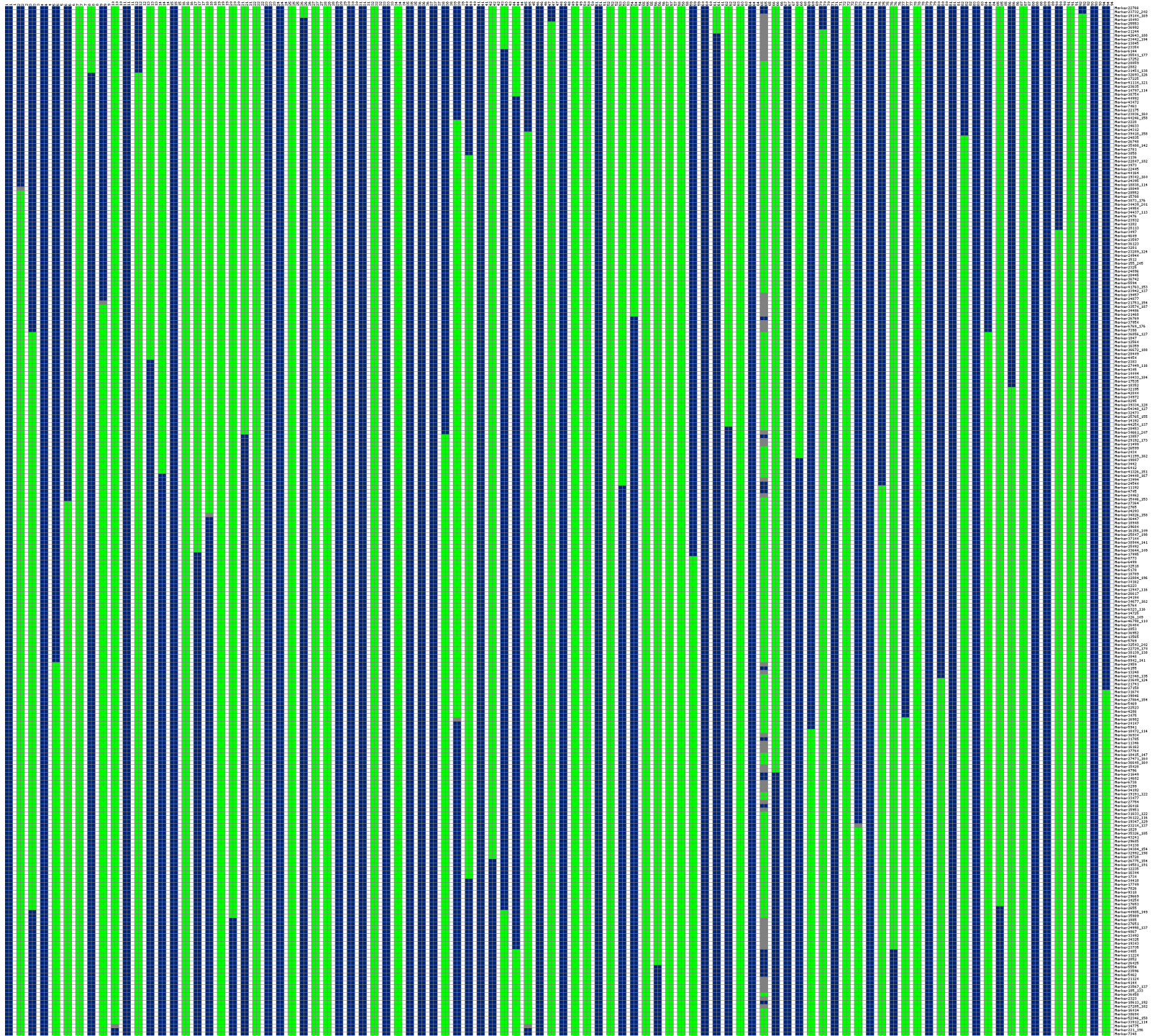



LG 10

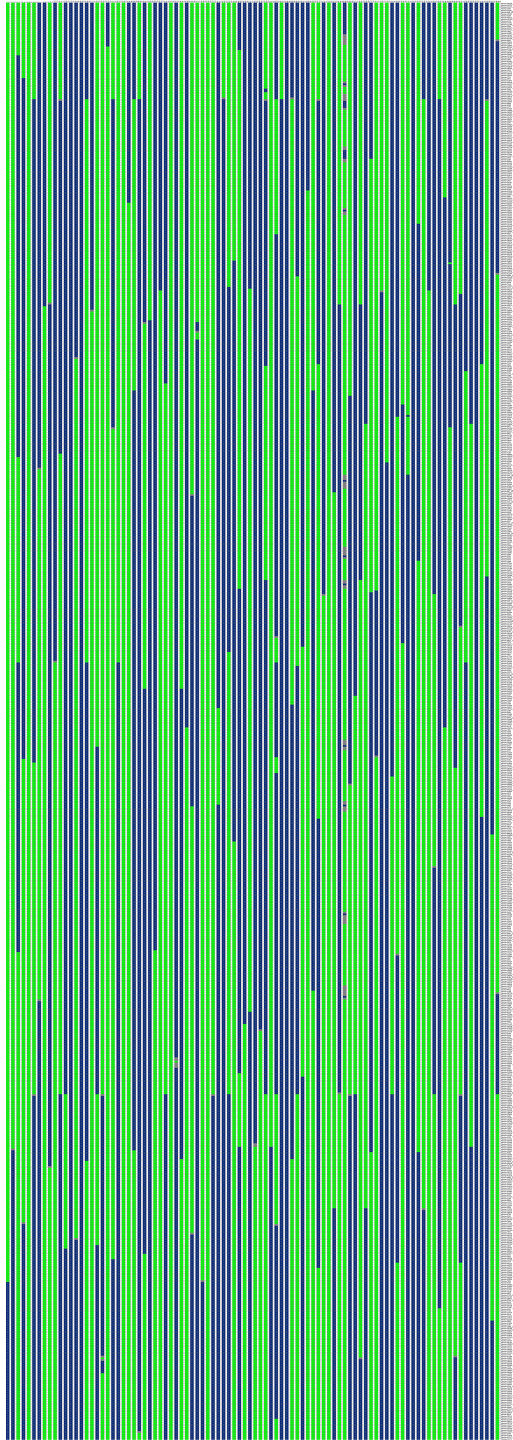

LG 11

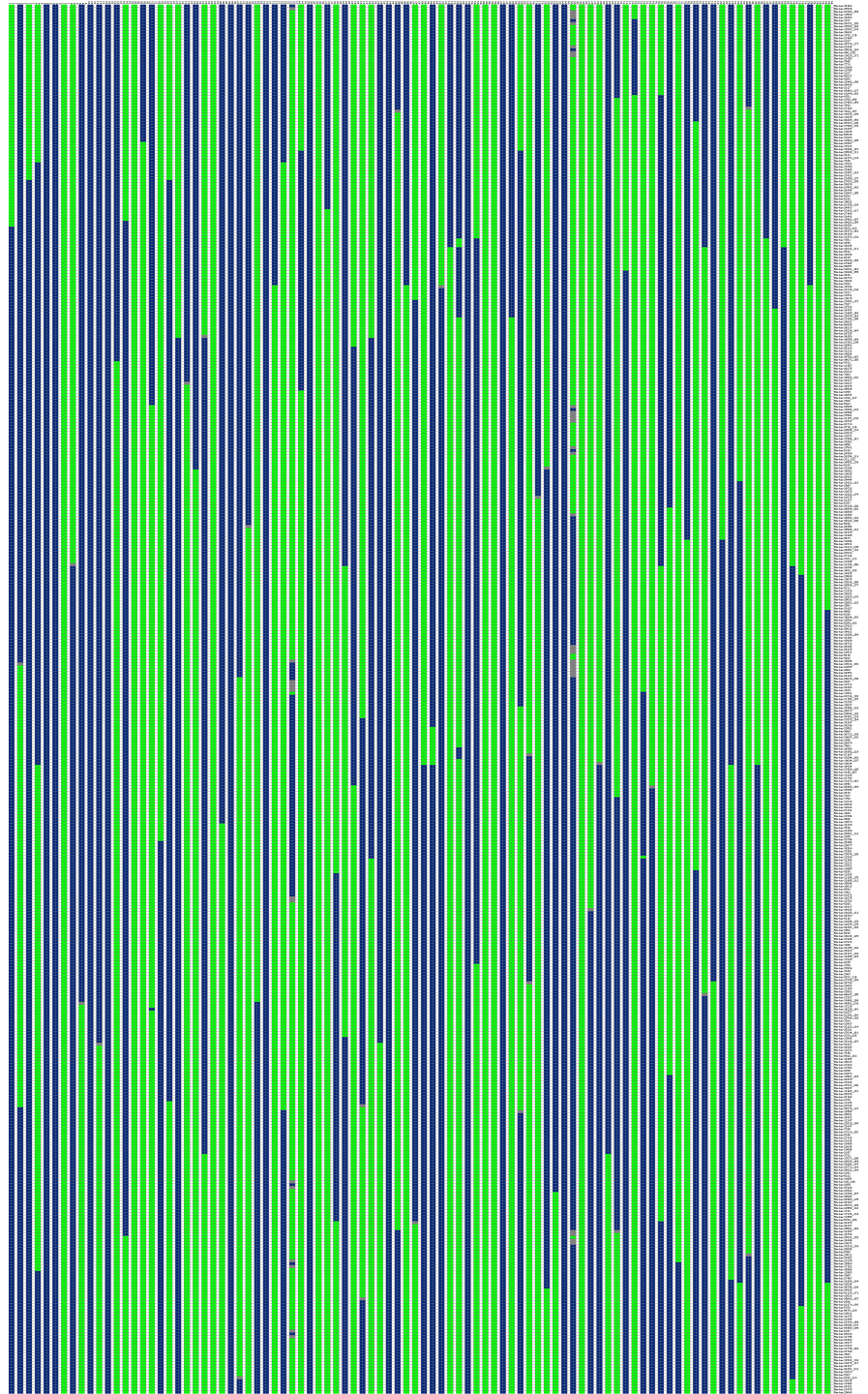

LG 12

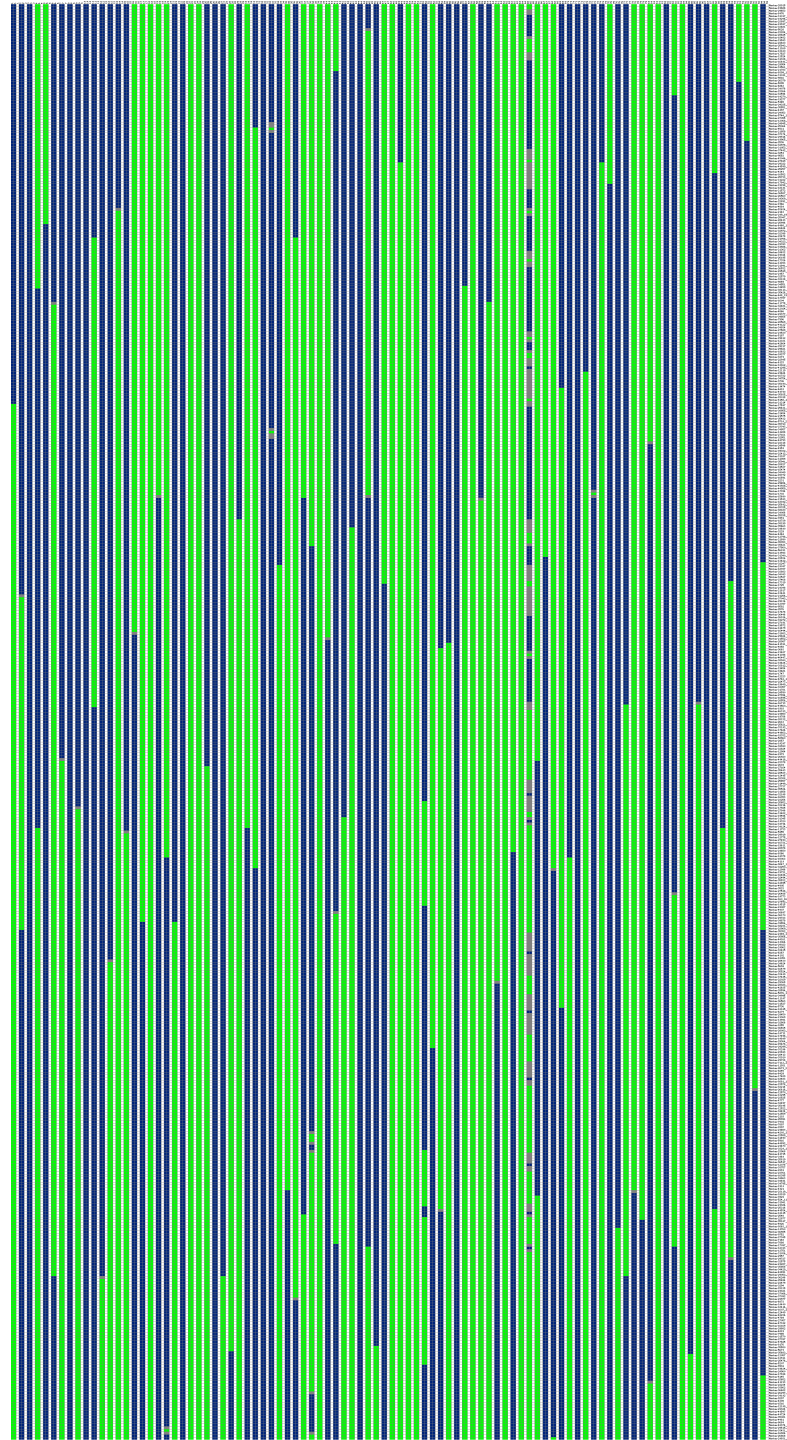

Supplement: FIGURE S2 — Haplotype map for each linkage group by each individual. Different individuals in rows were spaced by blank line, markers on the right were listed coordinate to the order in the linkage map. The swift of green and blue colors indicates the recombination events. The gray color represents missing marker. [file Image_2.PDF]
