# Supplementary material for: A Highly Dense Genetic Map for Ginkgo biloba Constructed Using Sequence-Based Markers
Source: Front Plant Sci. 2017 Jun 15;8:1041. doi: 10.3389/fpls.2017.01041 (PMC5471298; doi:10.3389/fpls.2017.01041)

LG 1

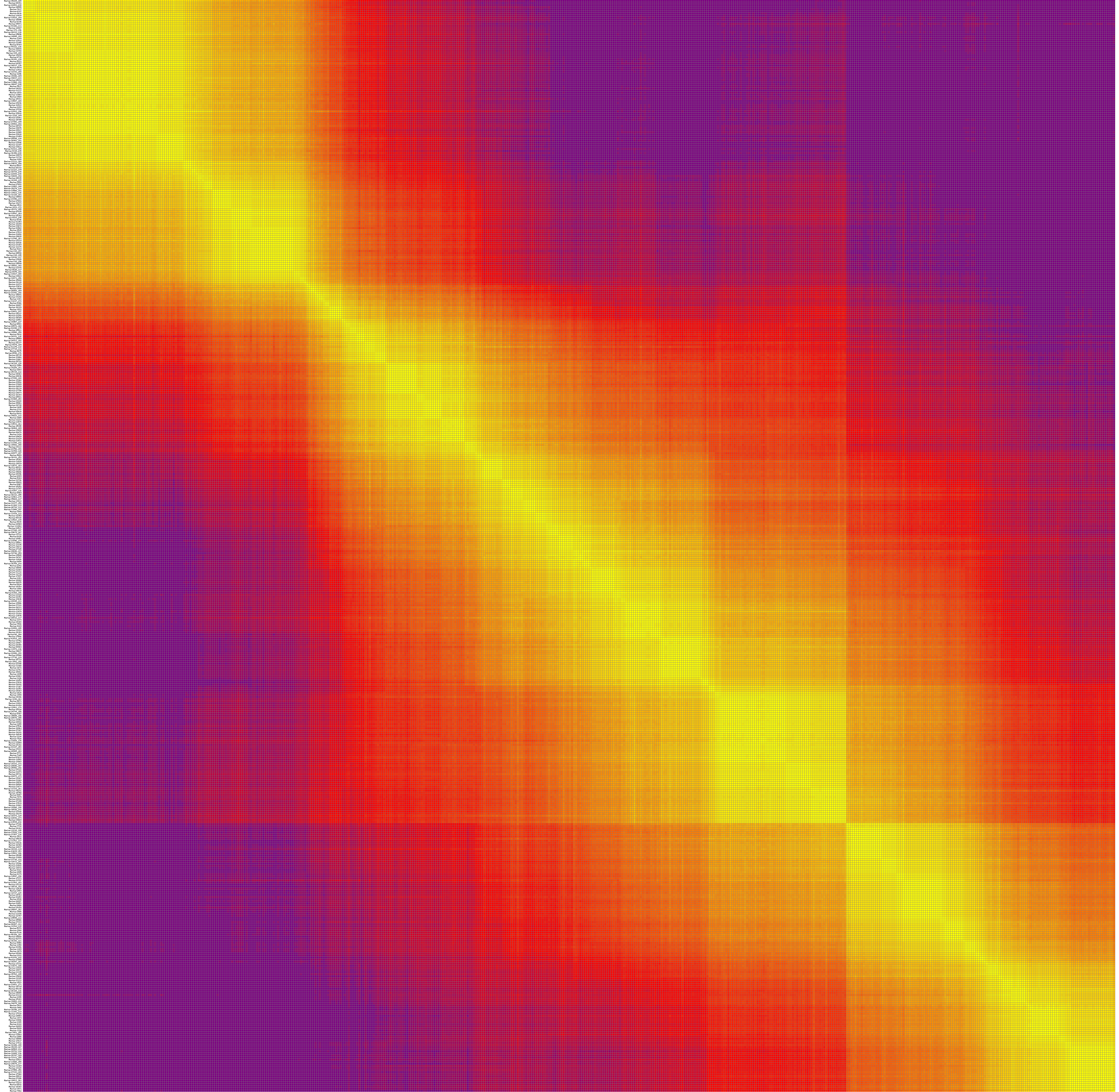

LG 2

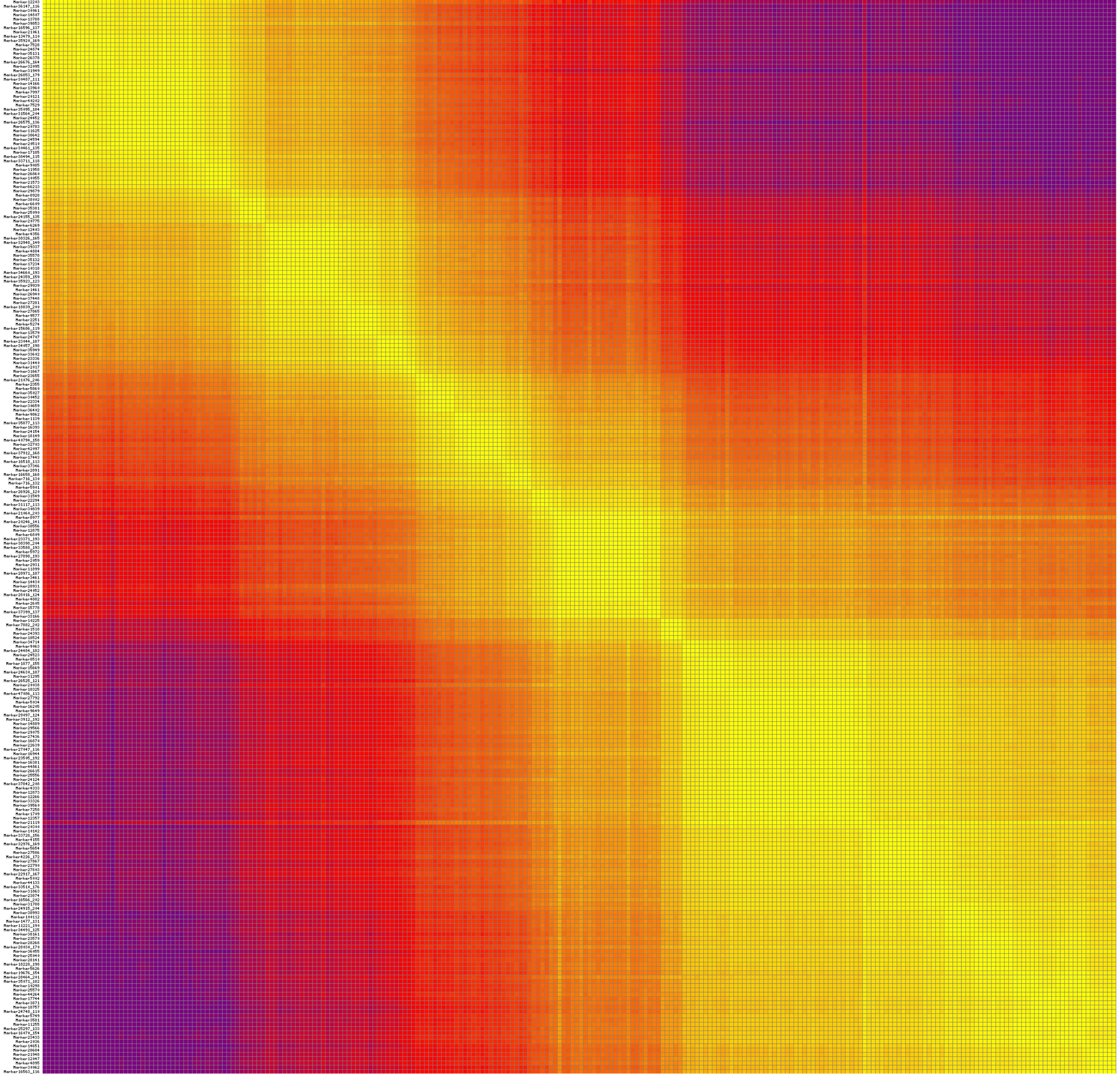

LG 3

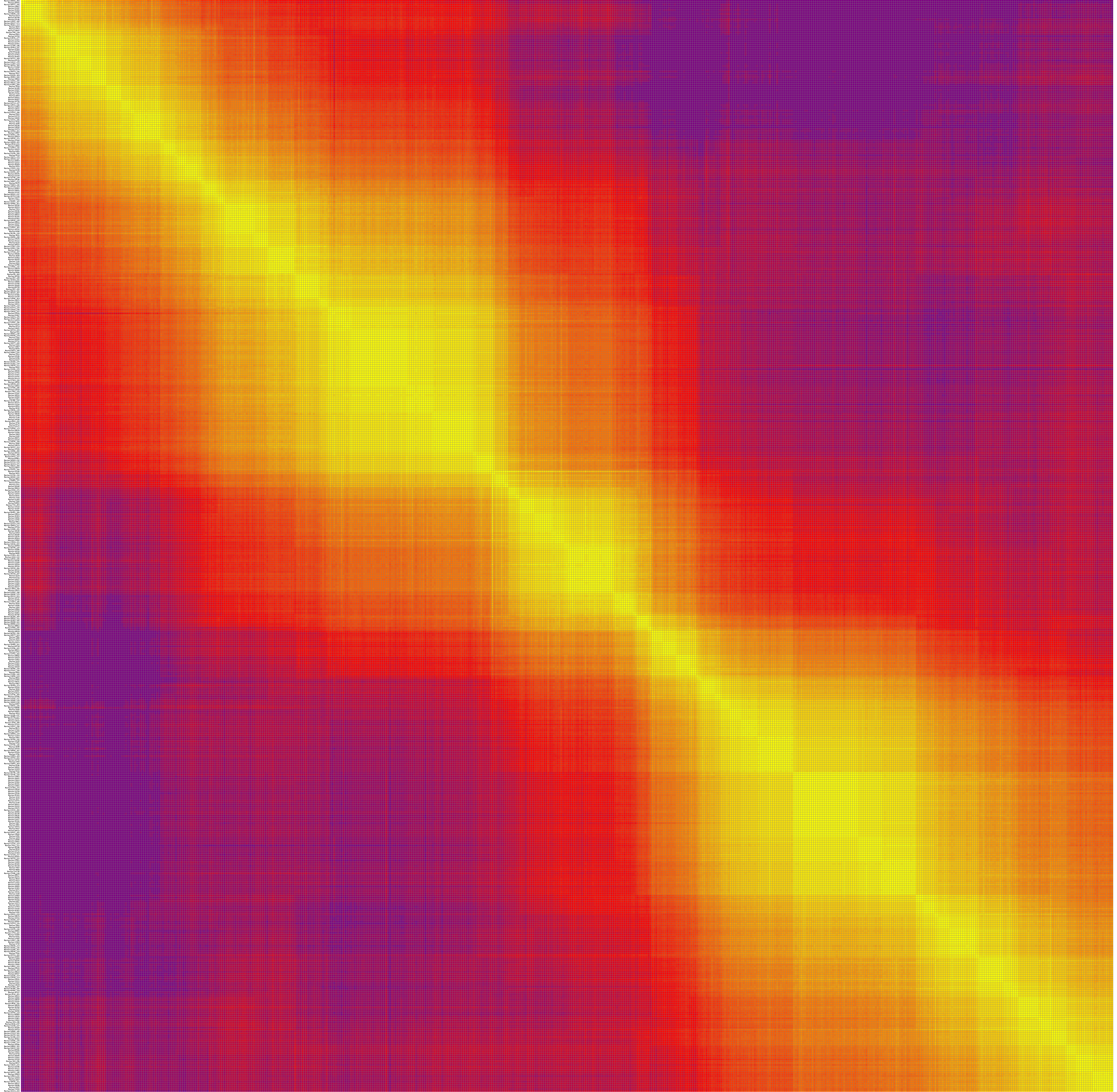

LG 4

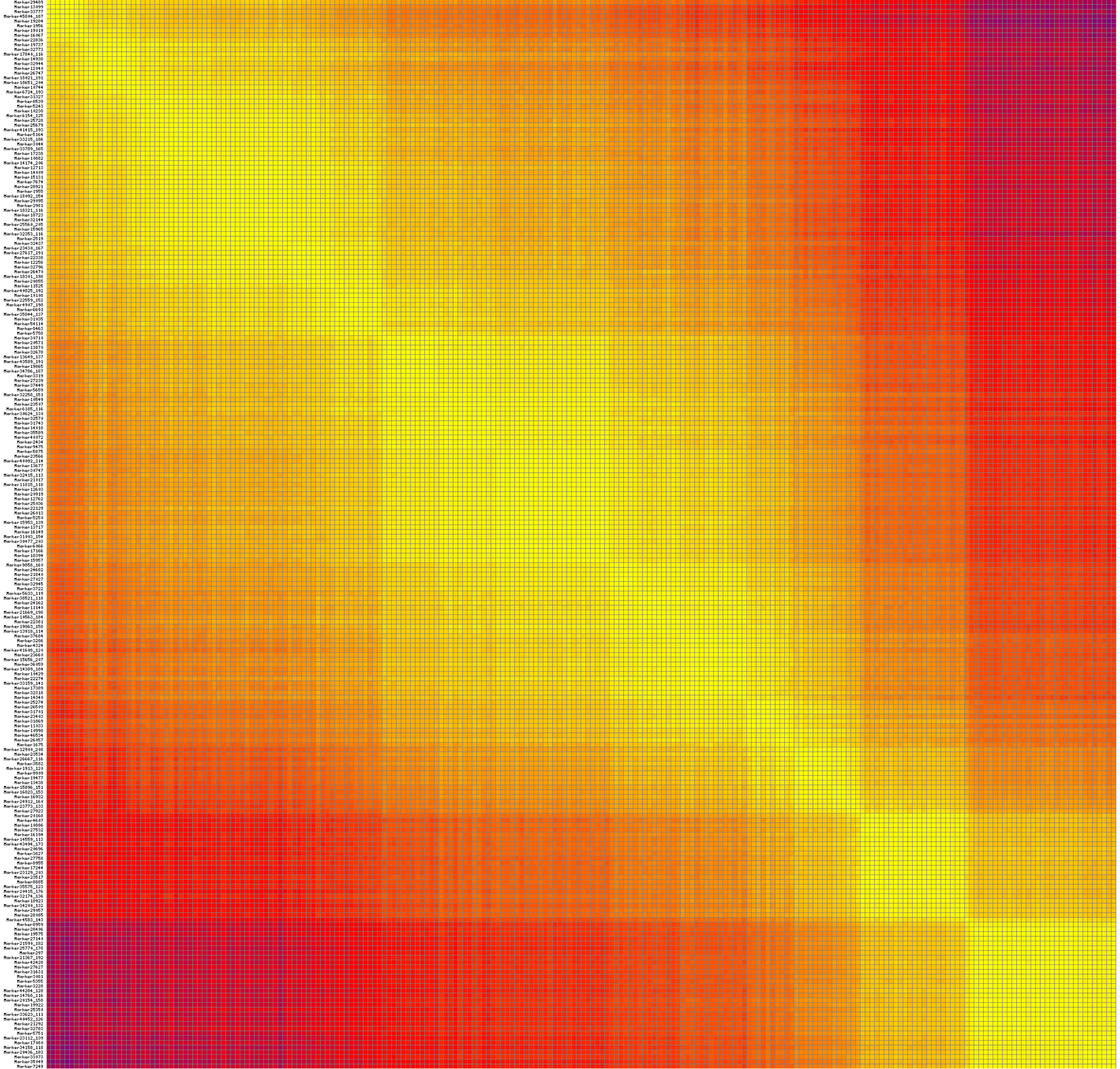

LG 5

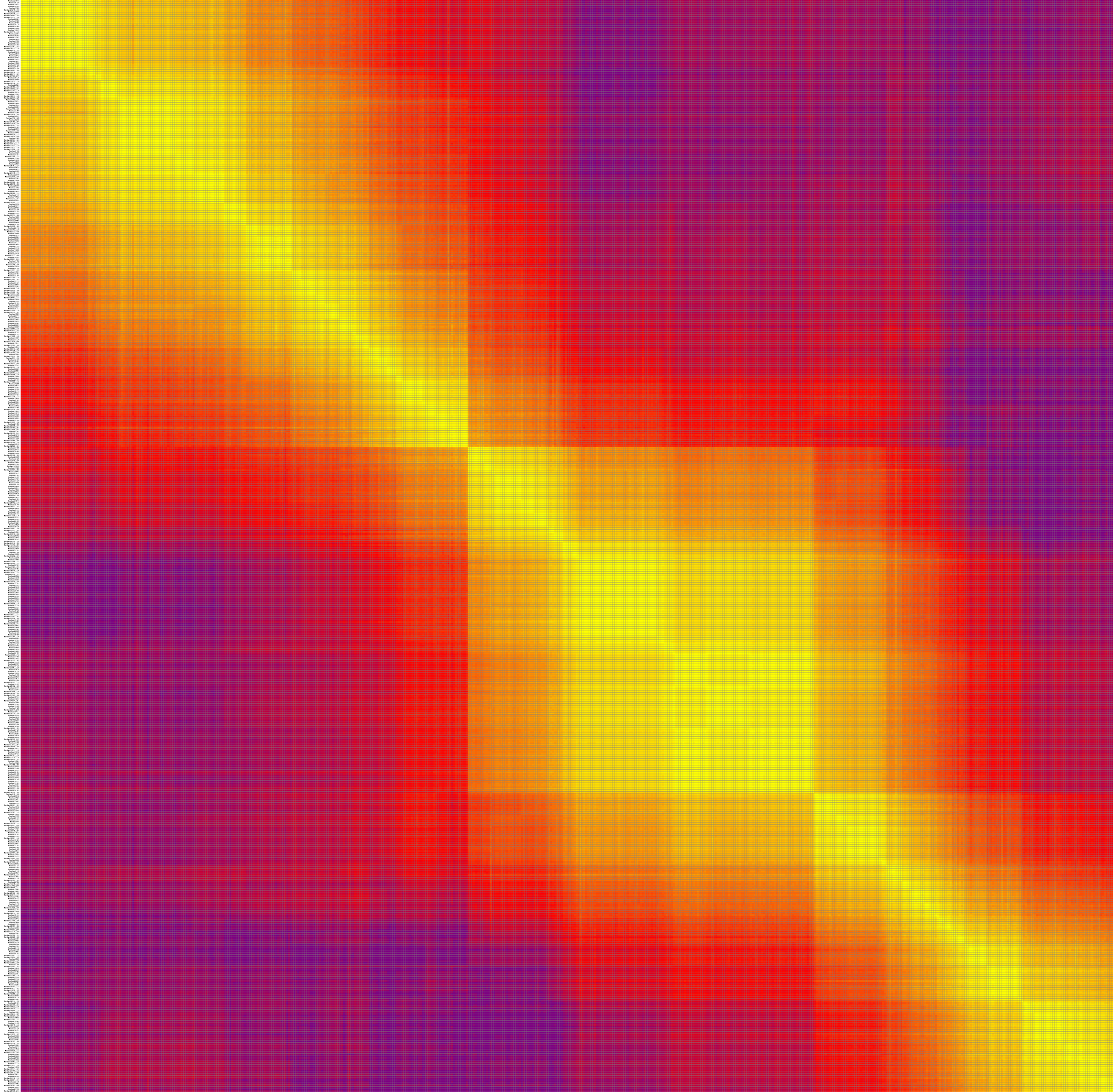

LG 6

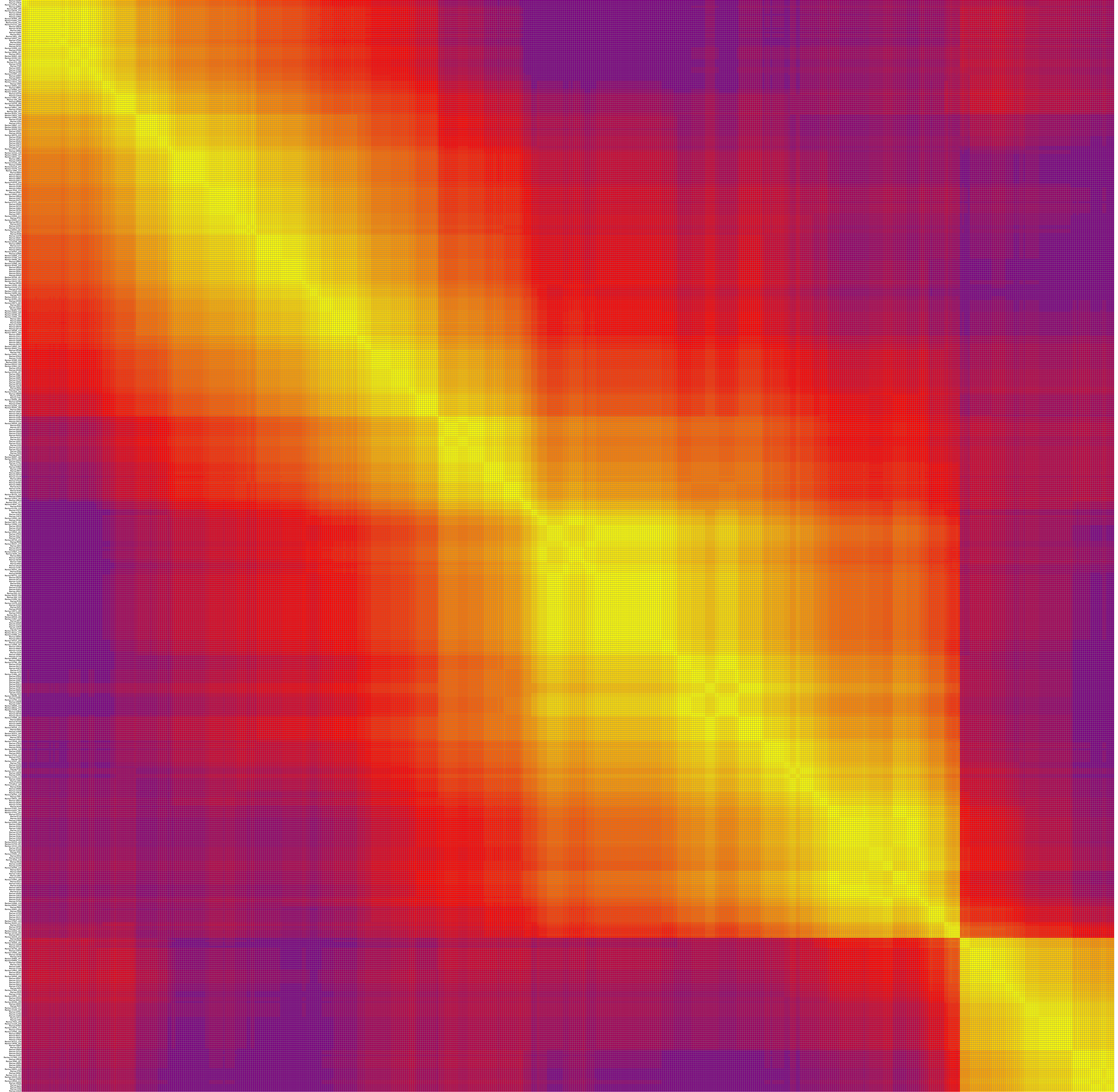

LG 7

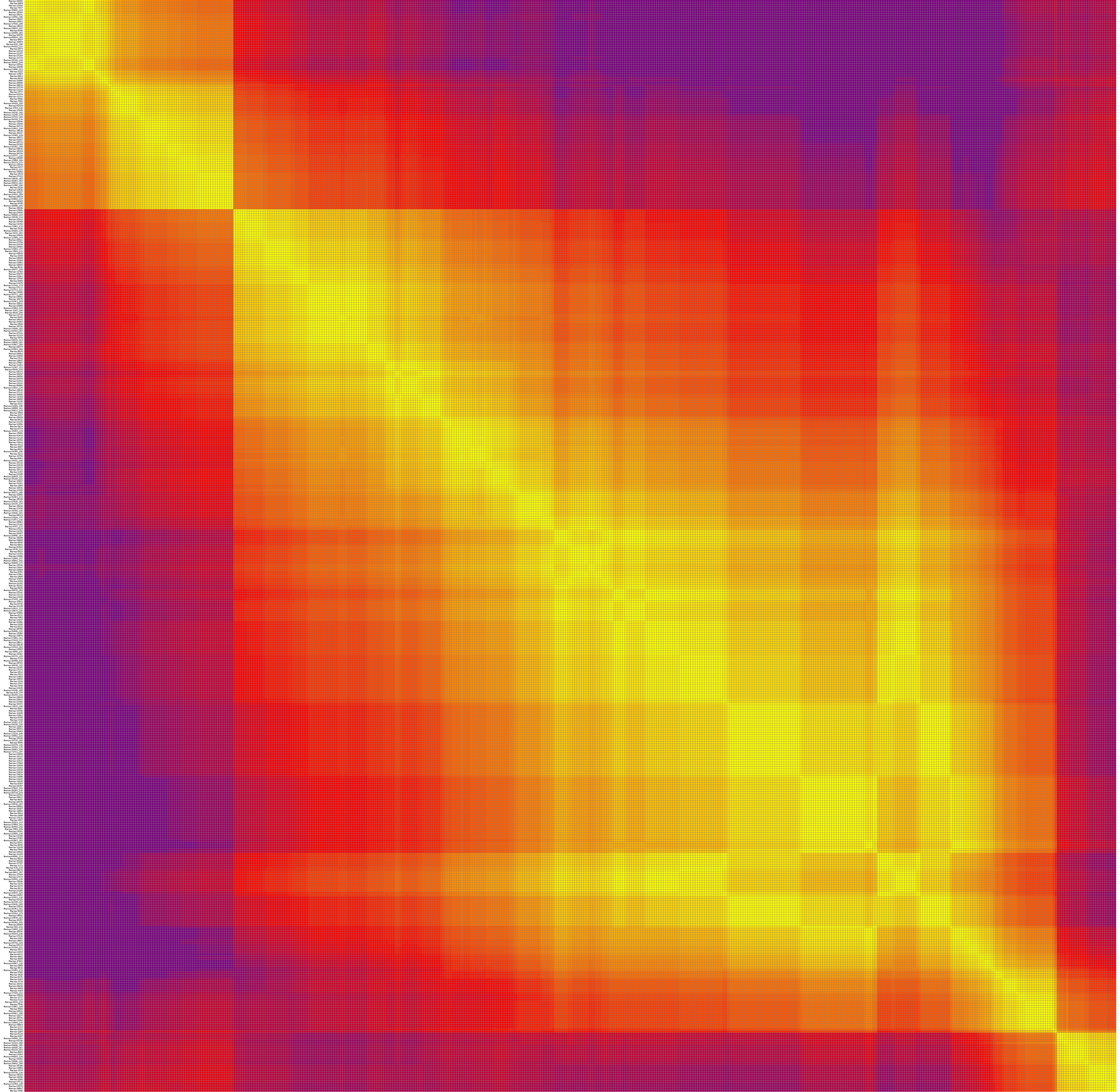

LG 8

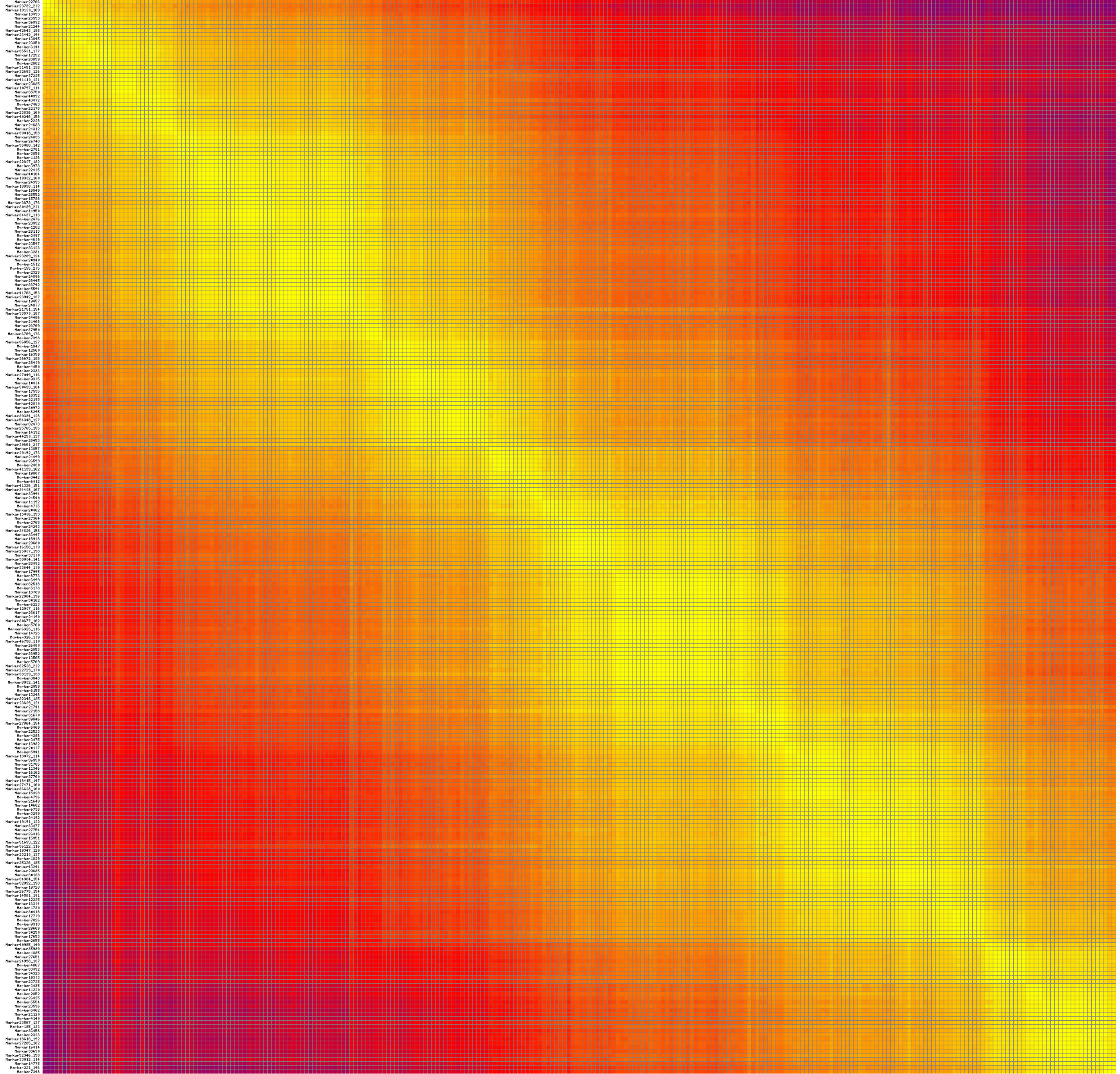

LG 9

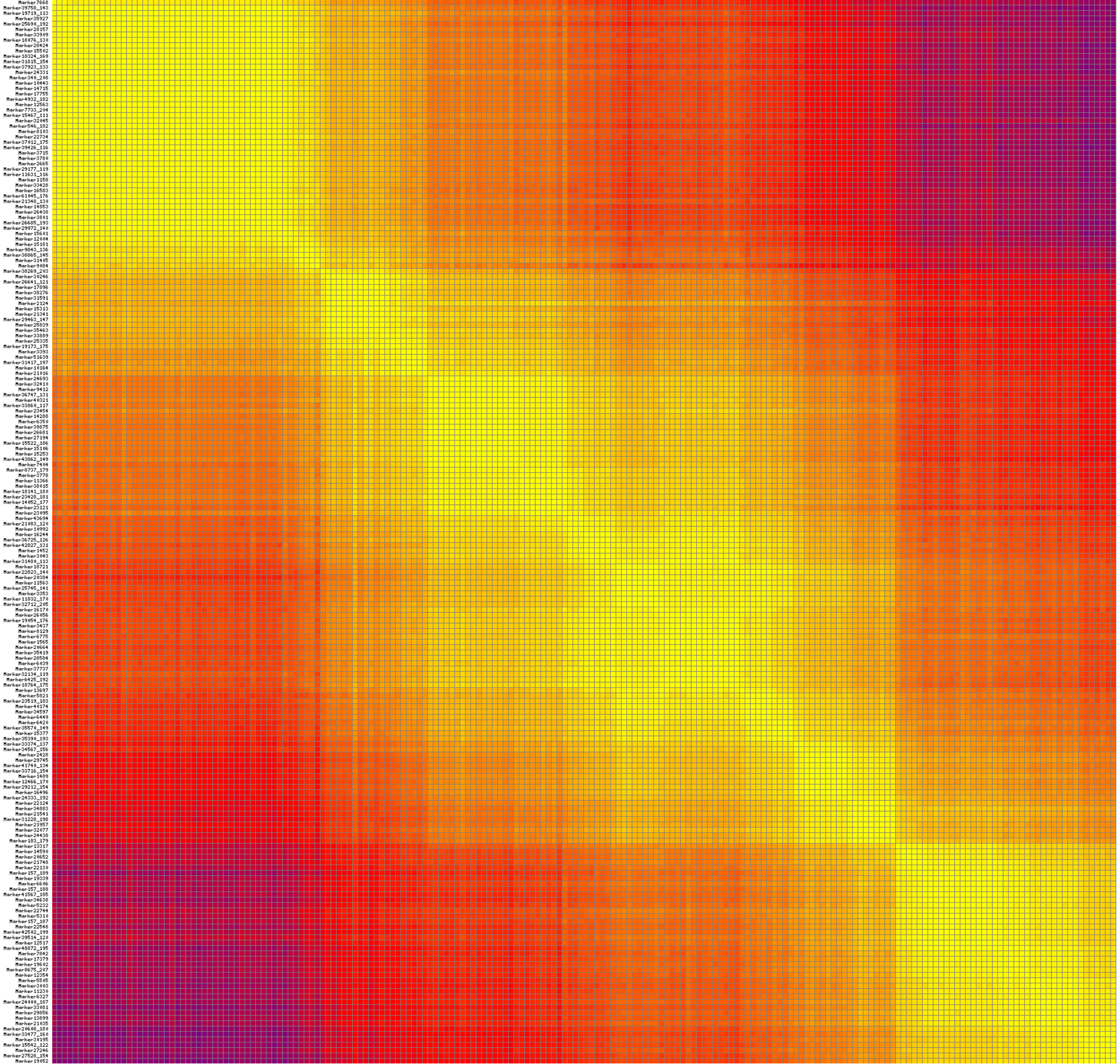

LG 10

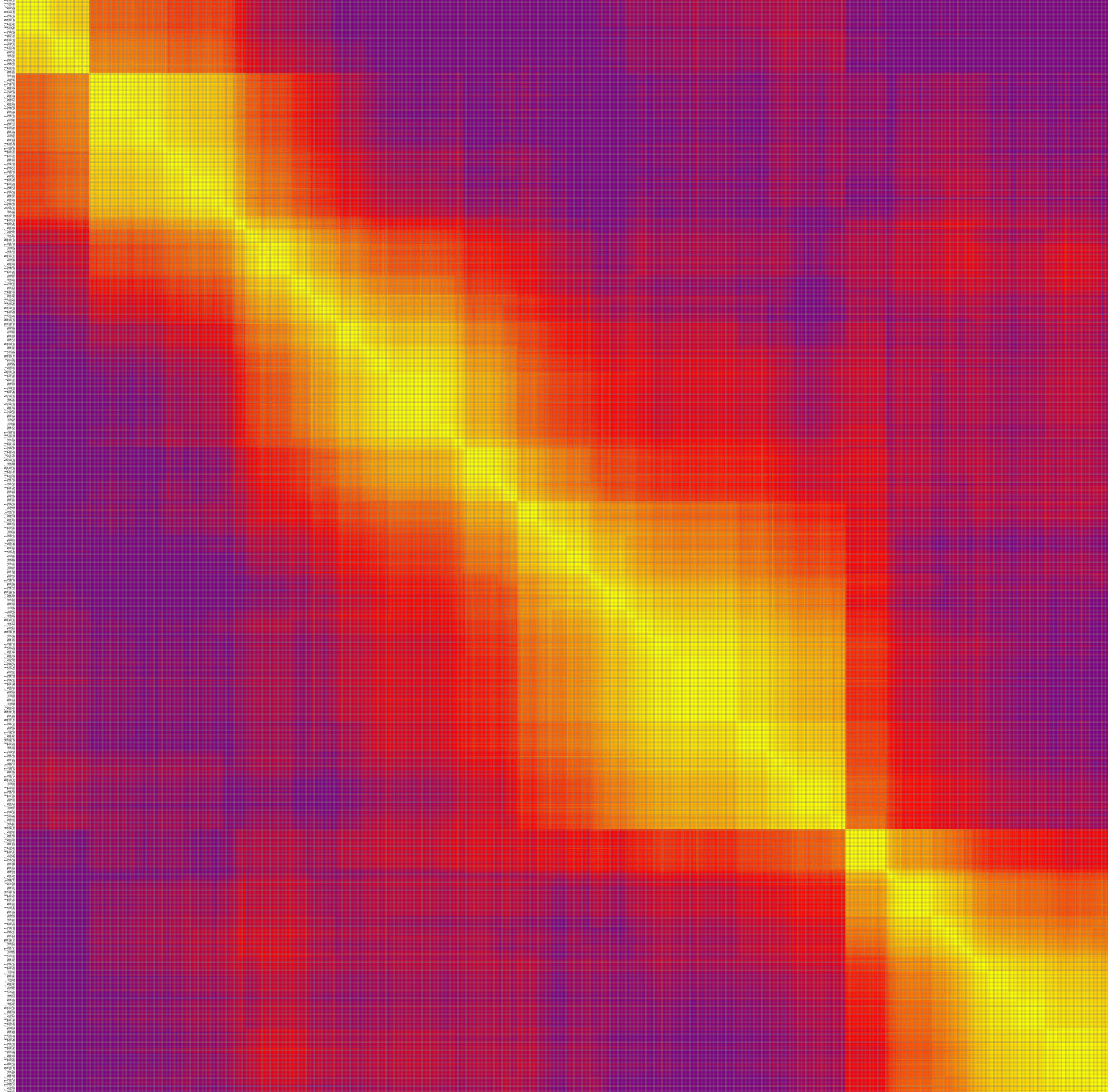

LG 11

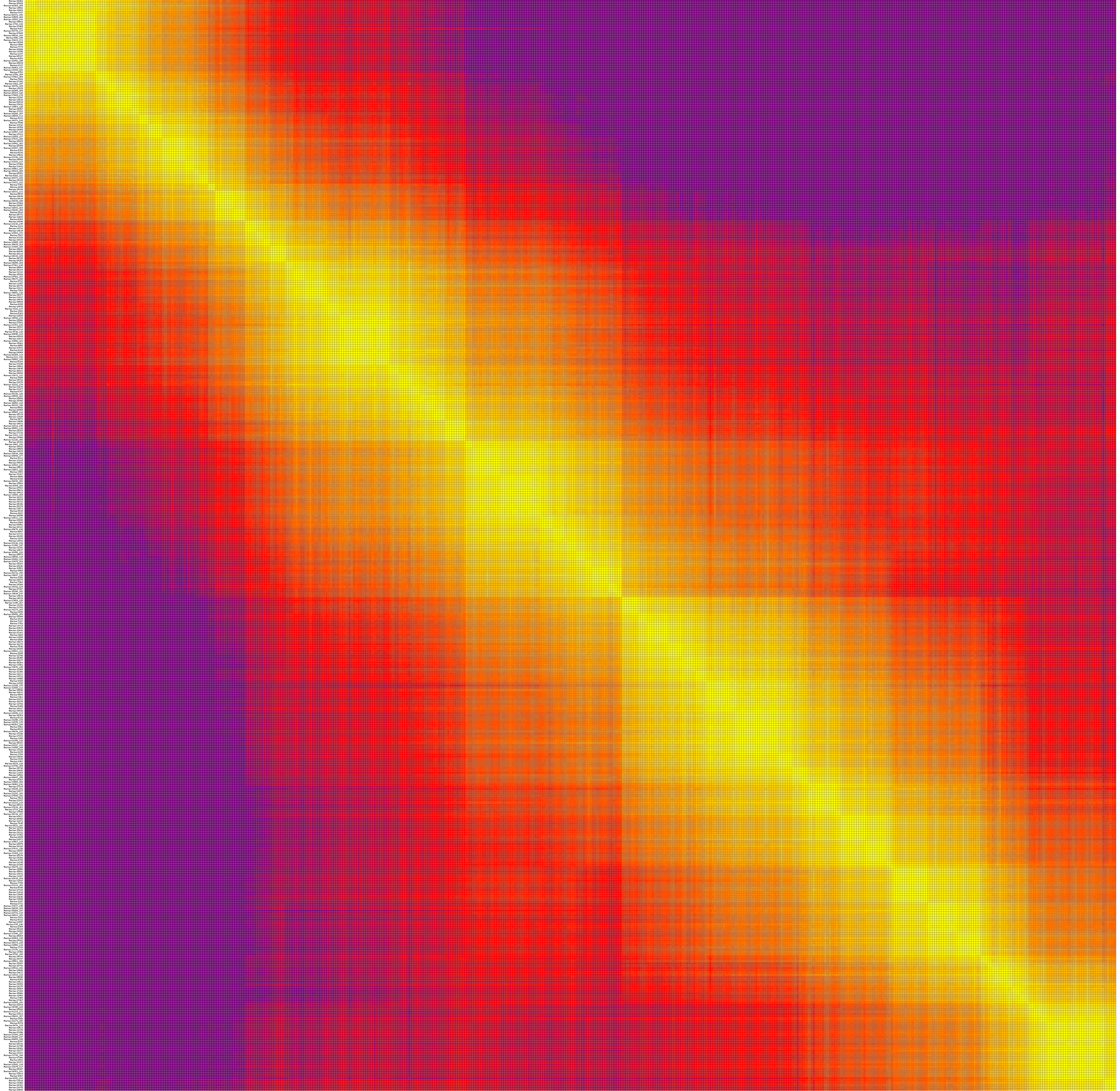

LG 12

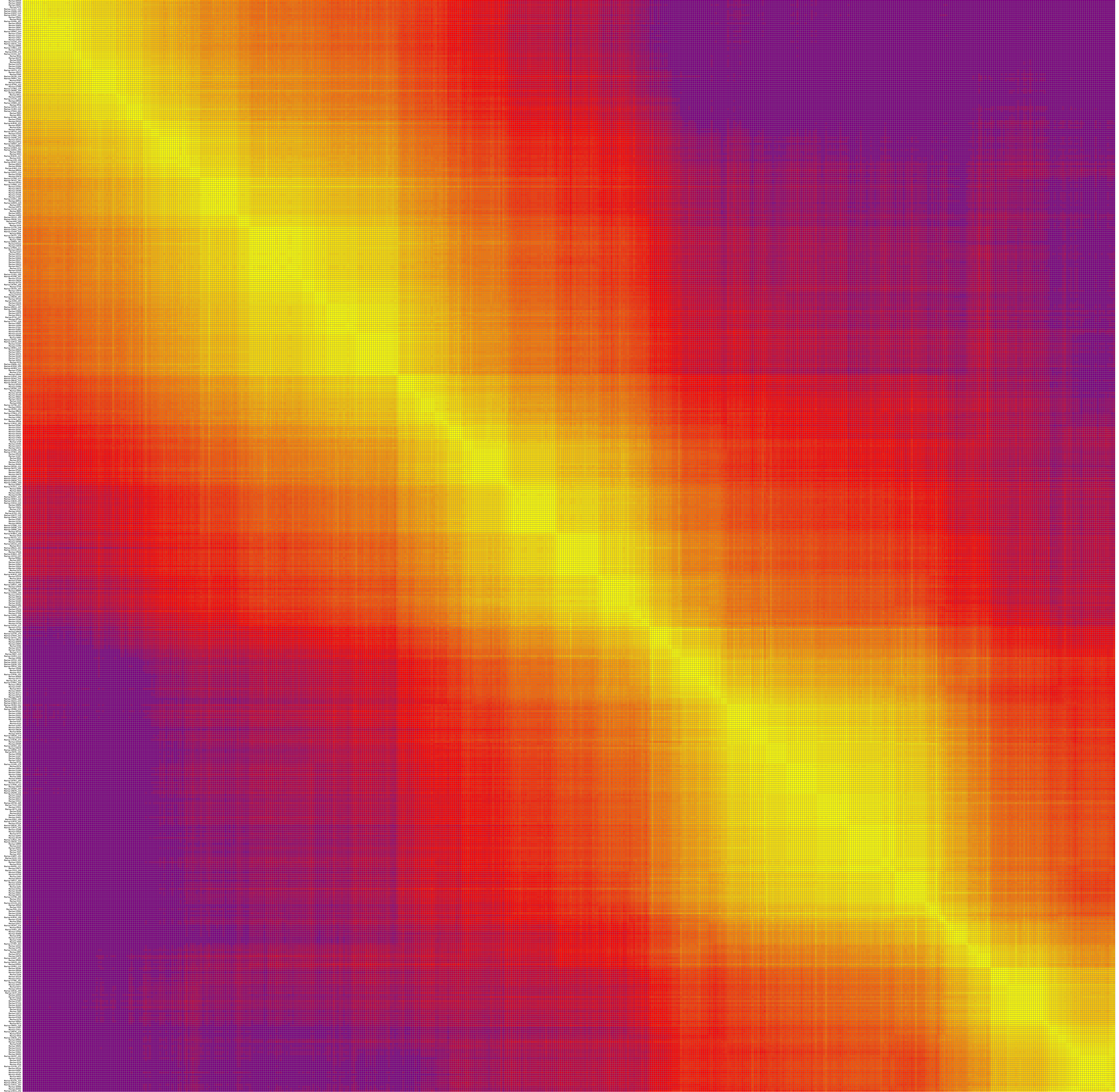

Supplement: FIGURE S3 — Heat map of each linkage group by pair-wise markers. Each cell represents the recombination rate of pair-wise markers. Yellow color indicates strong linkage, and changes of colors from yellow to purple indicating weaker linkage. [file Image_3.PDF]
